# Development of an organ failure score in acute liver failure for transplant selection and identification of patients at high risk of futility

**DOI:** 10.1371/journal.pone.0188151

**Published:** 2017-12-05

**Authors:** Francesco Figorilli, Antonella Putignano, Olivier Roux, Pauline Houssel-Debry, Claire Francoz, Catherine Paugam-Burtz, Olivier Soubrane, Banwari Agarwal, François Durand, Rajiv Jalan

**Affiliations:** 1 Liver Failure Group, UCL Institute for Liver and Digestive Health, UCL Medical School, Royal Free Campus, London, United Kingdom; 2 Hepatology and Liver Intensive Care Unit, Hospital Beaujon, APHP, Clichy, France; 3 Anaesthesiology-Intensive Care Unit, Hospital Beaujon, APHP, University Paris Diderot, Clichy, France; 4 Hepatobiliary-Pancreatic Surgery, Hospital Beaujon, APHP, Clichy, France; 5 Intensive Care Unit, Royal Free Hospital, Royal Free London NHS Foundation Trust, London, United Kingdom; University of Toledo, UNITED STATES

## Abstract

**Introduction:**

King’s College Hospital criteria are currently used to select liver transplant candidates in acetaminophen-related acute liver failure (ALF). Although widely accepted, they show a poor sensitivity in predicting pre-transplant mortality and cannot predict the outcome after surgery. In this study we aimed to develop a new prognostic score that can allow patient selection for liver transplantation more appropriately and identify patients at high risk of futile transplantation.

**Methods:**

We analysed consecutive patients admitted to the Royal Free and Beaujon Hospitals between 1990 and 2015. Clinical and laboratory data at admission were collected. Predictors of 3-month mortality in the non-transplanted patients admitted to the Royal Free Hospital were used to develop the new score, which was then validated against the Beaujon cohort. The Beaujon-transplanted group was also used to assess the ability of the new score in identifying patients at high risk of transplant futility.

**Results:**

152 patients were included of who 44 were transplanted. SOFA, CLIF-C OF and CLIF-ACLF scores were the best predictors of 3-month mortality among non-transplanted patients. CLIF-C OF score and high dosages of norepinephrine requirement were the only significant predictors of 3-month mortality in the non-transplanted patients, and therefore were included in the ALF-OFs score. In non-transplanted patients, ALF-OFs showed good performance in both exploratory (AUC = 0.89; sensitivity = 82.6%; specificity = 89.5%) and the validation cohort (AUC = 0.988; sensitivity = 100%; specificity = 92.3%). ALF-OFs score was also able to identify patients at high risk of transplant futility (AUC = 0.917; sensitivity = 100%; specificity = 79.2%).

**Conclusion:**

ALF-OFs is a new prognostic score in acetaminophen-related ALF that can predict both the need for liver transplant and high risk of transplant futility, improving candidate selection for liver transplantation.

## Introduction

Acetaminophen overdose (APAP-OD) is the most frequent cause of acute liver failure (ALF) in Western countries[1]. ALF is a life-threatening condition characterized by rapid severe liver injury and hepatic encephalopathy in patients without pre-existing liver disease. The clinical presentation is characterized by abnormal liver biochemical values, coagulopathy, decline in mental function, peripheral vasodilatation, features of the systemic inflammatory response syndrome and ultimately multi-organ failure (MOF) [2]. The period of active injury in acetaminophen overdose can be self-limiting and displays a hyperacute pattern in majority of patients; most of them recover with medical management alone including N-acetyl cysteine [3]. However, in patients who continue to deteriorate, an emergency liver transplantation (LT) is the only life-saving option and survival is inversely related to the time period elapsed between listing and the procurement of an organ.

The decision-making process for LT is currently based upon the King’s College Hospital criteria (KCH), which includes a set of parameters dedicated specifically to acetaminophen-induced ALF [4,5]. However, in a recent meta-analysis, KCH criteria, while showing a specificity of 95%, was associated with a very poor sensitivity (58%) in predicting LT-free mortality[6]. Several alternative prognostic scores have been developed with the aim to optimise sensitivity further while retaining specificity [7–9]. In addition to the difficulties faced with accurately identifying suitable LT candidates in ALF, it is important to recognise that the LT procedure itself is associated with high peri- and post-operative mortality and long-term complications, and requires life-long treatment with immunosuppression [10]. In the context of organ shortages and potential complications of LT, it is imperative that determining suitability of LT in ALF patients should also take in to account the probability of survival after LT in order to optimize organ allocation thus avoiding “futile” transplantation.

The primary aim of this study was to develop a prognostic score that would accurately predict the 3-month mortality in acetaminophen induced ALF (with or without LT) thus avoiding futile emergency LTs. Four different assessment strategies were used to address this hypothesis: 1) study best predictors of poor prognosis amongst the commonly used current scoring systems applied to patients with critical liver diseases; 2) to develop a new score using the best existing scores and additional clinical and biochemical variables, and validate its prognostic accuracy in an external cohort; 3) to characterize the pre-transplant features that may indicate high risk of futile LT; 4) to validate the accuracy of the new score in predicting futility of LT in an independent cohort.

## Patient and methods

### Study population and statistical analysis

We analysed all consecutive patients admitted with acetaminophen related ALF between 1990 and 2015 to the Intensive care unit of Royal Free Hospital (RFH), London (United Kingdom) and to the Liver Intensive Care Unit of Beaujon Hospital (BJH), Clichy (France). ALF was defined as the presence of severe liver injury with onset of hepatic encephalopathy (HE) within 12 weeks of the first symptoms[11]. Patients with pre-existing liver disease were excluded. Clinical and laboratory data were collected at the time of admission as well as the use of mechanical ventilation support, vasopressors and continuous renal replacement therapy. The study endpoint was all-cause mortality during the first 3 months after the admission. Patients were listed for liver transplantation according to KCH criteria [4] (the modified version including lactate levels was applied from 2002)[5]. Data for this study was obtained through archived patient notes in the hospital and the follow up data retrieved through a combination of follow up clinic notes, patient’s general physicians and direct telephone contact with patients themselves. This database is updated at regular intervals and has been analysed for other purposes previously. The research and development department at the Royal Free Hospital where this project was undertaken, have defined the study as a clinical audit and service evaluation project with no requirement for formal ethics approval. The data was fully anonymised and the need for consent was waived by the R&D department.

Continuous parametric variables were expressed as mean ± standard deviation and were compared using Student t-test. Non-parametric variables were showed as median and range, and compared with Kruskal-Wallis Test. Categorical variables were compared using the Chi-squared test. SPSS software package (version 20.0, SPSS Inc., Chicago, Ill, United States) and Medcalc® (version14.8.1, MedCalc Software bvba) were used for statistical analysis.

### Performance of existing scores

The principal liver-specific and general intensive care scores were calculated at time of admission. Those who received an emergency liver transplant (LT) were analysed separately from non-transplant (NOLT) patients. The KCH criteria were considered met in APAP-OD patients with pH<7.3 or lactate>3.5mmol/L following adequate resuscitation or the presence of following three features: international normalized ratio of Protrombin time (INR)>6.5, serum creatinine >3.4 mg/dl and a grade 3 or 4 hepatic encephalopathy based on West Haven criteria [12]. The Chronic Liver Failure Consortium Organ failure score (CLIF-C OF), with a range from 0 to 18, evaluates the failure of six organ systems (liver, kidney, brain, coagulation, circulation and respiratory system) taking into account the serum bilirubin, serum creatinine, INR, mean arterial blood pressure, PaO_2_ and fractional inspired concentration of oxygen (FiO_2_), PaO_2_/FiO_2_ ratio and the use of renal replacement therapy, vasopressors and invasive mechanical ventilation. Chronic Liver Failure Consortium Acute on Chronic Liver Failure (CLIF-C ACLF) score (range 0 to 100) is based on CLIF-C OF score incorporating additional variables of age and white blood cells count. Both scores were calculated using the CLIF research platform (www.clifresearch.com) to provide an estimate of the number of failed organs, the clinical severity, and the probability of death in the short and long-term follow up [13]. Model for end stage liver disease (MELD) score is based on total bilirubin, INR and serum creatinine and was calculated as 9.6 x log creatinine (mg/dL) + 3.8 x log bilirubin (mg/dL) + 11.2 x log INR + 6.43[14]. United Kingdom model for end-stage liver disease (UKELD) is a variant of MELD that include serum sodium [15] and is currently used to allocate organs in United Kingdom’s liver transplant list. The Sepsis-related Organ Failure Assessment (SOFA) provides an assessment of six organ systems: liver, renal, coagulation, cardiovascular, respiratory and central nervous system, the composite score ranging from 0 to 22 calculated on a 5 point grading scale (0 to 4) for each organ system [16]. Acute Physiology, Age, Chronic health Evaluation (APACHE) 2 (range 0–71) utilises the age of the patient, chronic health status, and a number of acute physiological variables including the worst value during the first 24 Hours of the heart rate, mean blood pressure, temperature, respiratory rate, PaO_2_, Alveolar-arterial gradient of Oxygen, haematocrit, white blood cells count, serum creatinine, presence of acute kidney failure, sodium, pH and Glasgow Coma Scale (GCS), [17]. APACHE 3 (range 0 to >299), additionally, also includes urine output, urea, glucose, total bilirubin, PaCO_2_ and a different grading of GCS parameters [18]. ROC curve analysis was used to assess the performance of prognostic scores to predict 3-month mortality. The cut-off was identified by Youden index and its Hazard Ratio (HR) was identified by Cox regression analysis. The Area Under the Curve (AUC), sensitivity, specificity, along with positive predictive value (PPV) and negative predicting value (NPV) and p value were determined.

### Developing a new score

The training cohort included NOLT patients admitted to RFH after 2001, the year that coincided with Norepinephrine as the preferred vasopressor of choice used in this setting. Multivariate Cox regression analysis was used to identify predictors of mortality between the first three best scores obtained from the previous analysis and the variables that were not part of them and resulted in significant association with mortality in univariate analysis. The factors showing a p<0.05 were used to develop the new score as follows: (regression coefficient β1) x (variable 1) + (regression coefficient β2) x (variable 2) + (regression coefficient β3) x (variable 3) + etc. The ability to predict the mortality of the new model was assessed and compared with the existing scores by ROC analysis. The new score was validated in the cohort of non-transplanted patients from BJH.

### Predictors of high risk of futility of LT

Patients from Beaujon hospital were used as explorative cohort. Futile LT was defined as occurrence of death within 48 hours of surgery in the context of development of MOF and/or irreversible brain damage but without major surgical complications (hepatic artery thrombosis, portal vein thrombosis, outflow obstruction, haemorrhagic shock) and/or primary graft non-function. The “non futile transplantation” included those who survived for at least 48 hrs following LT, and the deaths beyond this period were the direct result of transplant complications. Clinical and laboratory variables at the time of admission were analysed by Cox regression to identify the predictors of futility. The ability of the new score to predict futility was tested using ROC analysis.

## Results

### Baseline patients’ characteristics

**Fig 1:**
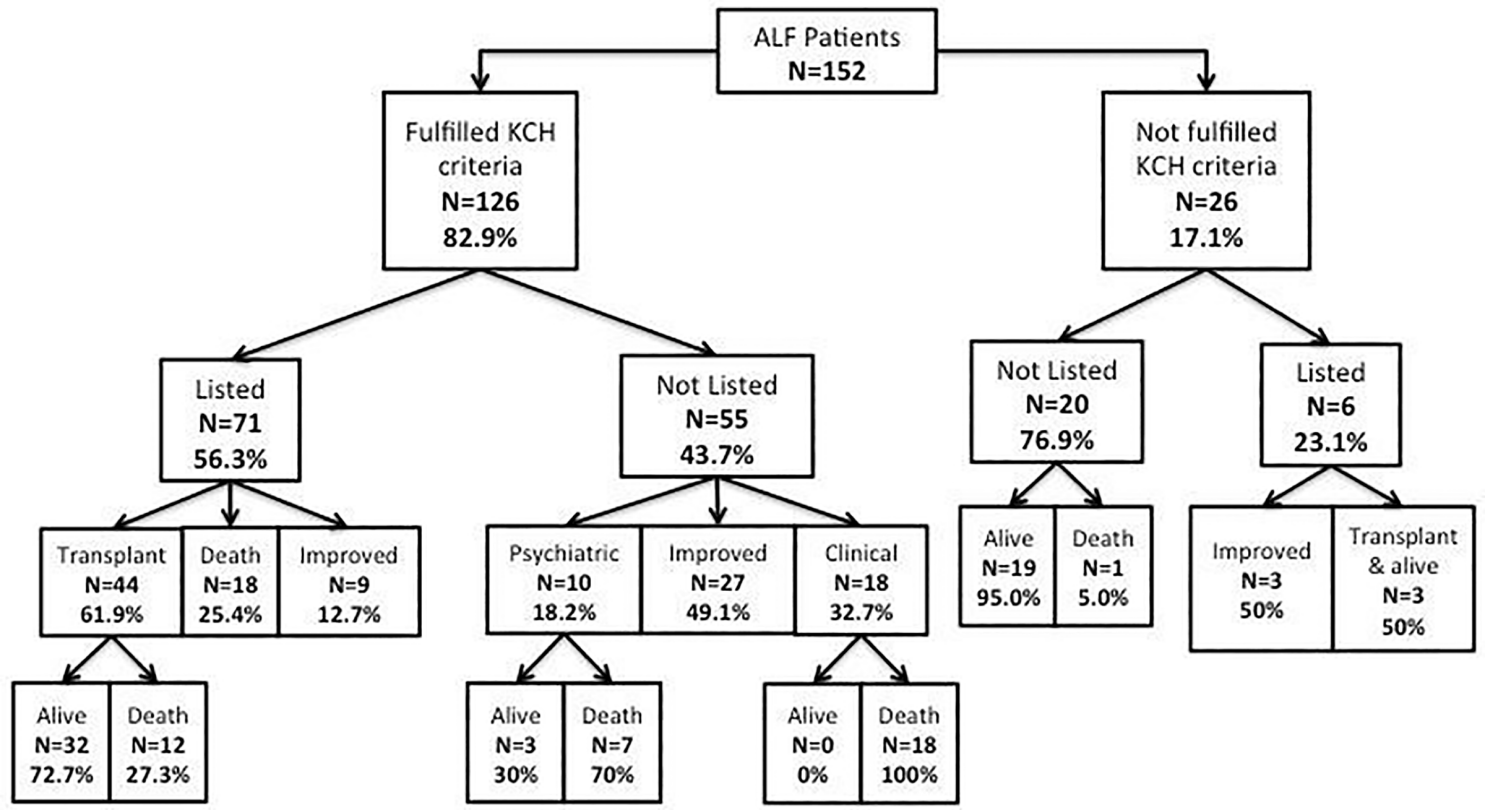
Flow chart of patients’ outcome in the study. Patients were first divided according to whether they fulfilled or not KCH criteria and therefore were listed or not for liver transplantation. Reasons for liver transplant ineligibility and the outcome of the different categories were reported. ALF: Acute Liver Failure; KCH: Kings College criteria.

152 patients admitted with acetaminophen related ALF were included in this study. As shown in Fig 1, 126 (82.9%) patients met the KCH criteria. Of them, 71 (56.3%) were listed for an emergency LT: 18 (25.4%) died on the waiting list, 9 (12.7%) improved without an LT and 44 (61.9%) were transplanted. (**Fig 1).** The mortality rate after 3 month from the hospitalization was 27.3%. Despite meeting the indication for an emergency LT, 18 (32.7%) were considered too sick to receive LT, 10 (18.2%) had psychiatric contraindication and 27 (49.1%) recovered spontaneously. Among patients who did not fulfil KCH criteria (n = 26/152, 17.1%), 6 (23.1%) were listed for LT: 3 were transplanted and survived at 3 months, while the other 3 improved and were removed from the waiting list. Of the 20 patients who did not fulfil KCH criteria and were not listed, only 1 died after 5 days due to MOF. As shown in Table 1, 98 patients were admitted to RFH and 54 to BJH. There were no differences between the two cohorts regarding the age, gender, 3-month mortality and fulfilment of KCH criteria during hospitalization (S1 Table).

**Table 1:** Baseline characteristics of population study.

	RFH (n = 98)		BJH (n = 54)		p value
**Age (years)**	37.6	±13.4	39.3	±13.4	NS
**Sex (Female)**	65	66.3%	35	64.8%	NS
**KCH (yes)**	83	84.7%	43	79.6%	NS
**LT**	19	19.4%	28	51.9%	**<0.001**
**3-months mortality**	38	38.8%	18	33.3%	NS
**Days to death from admission**	8	0–92	1	0–52	**0.002**
**Grade HE 3–4**	63	64.3%	36	66.7%	NS
**Vasopressor use**	60	61.2%	26	48.1%	NS
**Renal replacement therapy**	61	62.2%	31	57.4%	NS
**Mechanical ventilation**	66	67.3%	29	53.7%	NS
**Body temperature (°C)**	36.5	31–39.2	36.9	32.9–39.0	NS
**Heart rate (bpm)**	120	32–165	110	60–160	**0.003**
**Mean arterial pressure (mmHg)**	63	43–97	73	35–123	**0.002**
**PCO2 (kPa)**	4.5	2–8.7	3.9	1.3–6.0	**<0.001**
**PaO2 (kPa)**	13.8	6.3–36.7	15.4	6.9–35.3	NS
**FiO2 (%)**	30	21–100	21	21–100	NS
**PaO2/FiO2 (kPa)**	50	12.8–110.2	53	8.3–151	NS
**A-a Gradient (mmHg)**	65.1	-79.2–563.2	39.2	-88.3–636	NS
**Norepinephrine dose (mcg/min)**	0.0	0–130	15	0–265.6	**0.004**
**Ammonia (umol/L)**	98	35–286	300	50–999	**<0.001**
**Platelets (10^9/L)**	80.5	6–288	121.5	9–461	**<0.001**
**Creatinine (umol/L)**	226.5	43–825	145.5	49–564	NS
**Urea (mmol/L)**	8	1.8–46.9	5.7	1.2–17.8	**0.005**
**Total Bilirubin (umol/L)**	88	3–581	67.5	8–492	NS
**INR**	8	1.2–16	6.9	2.2–11.9	NS
**pH**	7.34	6.93–7.51	7.36	6.92–7.52	NS
**Sodium (mmol/L)**	129.8	± 7.22	136.7	±6.21	NS
**HCO3 (mmol/L)**	19.1	±5.22	15.6	±5.80	NS
**Lactate (mmol/L)**	4.8	0.8–35.8	8.6	1.1–24.9	**0.009**

Variables are expressed as numbers and percentages or mean ± standard deviation (or median and range when appropriate). KCH, King’s College Hospital criteria; LT, liver transplantation; HE, hepatic encephalopathy; INR, international normalized ratio.

However, more patients in the BJH cohort received LT than in RFH (51.9% vs 19.4%; p<0.001) and the median time to death from admission was significantly shorter (1 vs 8 days; p = 0.002). The BJH cohort also showed higher levels of serum ammonia (300 vs 98 umol/L; p<0.001), lactate (8.6 vs 4.8 mmol/L; p = 0.009) and norepinephrine requirement (15 vs 0 mcg/min; p = 0.004). No statistically significant differences were seen in the grade of HE and the use of organ support therapy in the first 24 hours of hospitalization.

### Performance of existing scores

We analysed the 98 patients admitted in the intensive care unit of RFH. The mean age was 37.6±13.4 years and 66.3% were female. 64.3% presented with severe grades (3/4) of hepatic encephalopathy. An emergency LT was performed in 19/98 (19.3%) patients, all of them had met the King’s College Hospital criteria. Of 79 NOLT patients, 13 were listed but did not receive a LT. Eight of them (61.5%) died on the waiting list and 5 (38.5%) spontaneously recovered. Of the 66 patients not listed, 16 (24.2%) did not fulfil KCH criteria; the remaining 50 patients met KCH criteria but LT was contraindicated in 15 patients (22.7%) who were too sick to receive an LT and 10 (15.2%) patients had psychiatric contraindication; 25 recovered spontaneously. As shown in Table 2, significantly higher number of LT patients needed mechanical ventilation (89.5% vs 62%; p = 0.022) than NOLT patients. The 3-month mortality rate was 37.8% and there was no difference between transplanted and non-transplanted patients (LT 36.8%, NOLT 39.2%, p = 0.847). All of death within 3 months among NOLT patients were due to MOF and only the 22.6% had culture-positive sepsis. In transplant patients 5 (71.4%) died due to sepsis, 1 (14.3%) due to MOF without sepsis and 1 (14.3%) committed suicide after 88 days from liver transplant. The survival time between the admission and death was significantly longer in the LT group (34 (5–92) vs 5.5 (0–21) days; p<0.001). The median MELD score and mean CLIF-C ACLF score were significantly higher in the LT cohort.

**Table 2:** Comparison between not-transplanted (NOLT) and transplanted (LT) patients (RFH cohort).

	NOLT (n = 79)		LT (n = 19)		p value
**Age (years)**	39.2	±13.6	31.4	±11.0	NS
**Sex (Female)**	49	62.0%	16	84.2%	NS
**KCH (yes)**	64	81.0%	19	100%	**0.039**
**3-month mortality**	31	39.2%	7	36.8%	NS
**Days to death from admission**	5.5	0–21	34	5–92	**<0.001**
**Grade HE 3–4**	51	64.6%	12	63.2%	NS
**Vasopressor use**	46	58.2%	14	73.7%	NS
**Renal replacement therapy**	48	60.8%	13	68.4%	NS
**Mechanical ventilation**	49	62.0%	17	89.5%	**0.022**
**MELD**	33.8	8–44	36.6	29–43	**0.002**
**UKELD**	70	45–82	71	63–81	NS
**APACHE 2**	23	±8.76	27.6	±7.16	NS
**APACHE 3**	125	±32.2	140	±27.9	NS
**SOFA**	11	3–20	12	3–20	NS
**CLIF-C OF**	13	9–17	14	10–17	NS
**CLIF-C ACLF**	51.2	±13.4	54.7	±6.81	**0.029**

Variables are expressed as numbers and percentages or mean ± standard deviation (or median and range when appropriate).

KCH, King’s College Hospital criteria; HE, hepatic encephalopathy; MELD, model for end-stage liver disease; UKELD, United Kingdom model for end-stage liver disease; APACHE, acute physiology in chronic health evaluation; SOFA, Sequential organ failure assessment; CLIF-C OF, Chronic liver failure-consortium organ failures; CLIF-C ACLF, Chronic liver failure-consortium acute on chronic liver failure.

As shown in Table 3, in the NOLT cohort, all of the scores calculated at the time of admission had statistically significant ROC values. The KCH criteria had the lowest AUC (0.638) and the lowest PPV (49%) and prediction of poor outcome (the 3-month mortality) associated with a sensitivity of 83.9% and specificity of 43.7%. The highest sensitivity (100%) belonged to APACHE 3 but it was associated with the lowest specificity (41.6%) that resulted in an AUC of 0.740. The best score was SOFA with an AUC of 0.799 followed by CLIF-C OF (0.793) and CLIF-C ACLF (0.762). CLIF-C OF showed a higher sensitivity than SOFA (93.5% vs 77.4%) and a lower specificity (58.3% vs 70.8%). There was no significant difference between these two AUCs (p = 0.849) and all of them were significantly higher when compared to KCH AUC (SOFA vs KCH p = 0.009; CLIF-C OF vs KCH p = 0.012). SOFA cut-off (11) was associated with a HR of 5.2 obtained by Cox univariate analysis (95% I.C. 2.13–12.73; p<0.001) and showed a PPV of 63.1% and a NPV of 82.9%. Patients with a CLIF-C OF score higher than the cut off (12) had a 38.9-fold increase in mortality risk (95% I.C. 1.66–907.57, p = 0.023). The CLIF-OF positive predictive value was 59.1% and the chance of a patient to survive with a CLIF-C OF score less than 12 (NPV) at the admission was 93.2%. In the LT group none of the scores calculated at the time of admission had statistically significant at ROC values in predicting the 3-month mortality from admission.

**Table 3:** Performance of main prognostic scores in predicting 3-month mortality in not-transplanted (NOLT) and transplanted (LT) patients (RFH cohort).

**NOLT**	**AUC**	**p**	**cut-off**	**HR**	**Sensitivity**	**Specificity**	**PPV**	**NPV**
**MELD**	0.671	**0.007**	33.4	2.595	74.2	54.2	51.1	76.5
**UKELD**	0.674	**0.006**	71	2.127	54.8	72.9	56.5	71.4
**APACHE 2**	0.757	**<0.001**	18	13.572	96.8	45.8	53.5	95.6
**APACHE 3**	0.740	**<0.001**	101	35.870	100	41.6	52.4	100
**SOFA**	**0.799**	**<0.001**	**11**	**5.2**	**77.4**	**70.8**	**63.1**	**82.9**
**KCH**	0.638	**0.005**	y	3.28	83.9	43.7	49	80.8
**CLIF-C OF**	**0.793**	**<0.001**	12	38.914	93.5	58.3	59.1	93.2
**CLIF-C ACLF**	0.762	**<0.001**	46	9.059	93.5	52.1	55.7	92.5
**LT**	**AUC**	**p**	**cut-off**	**HR**	**Sensitivity**	**Specificity**	**PPV**	**NPV**
**MELD**	0.564	NS	40.7	2.952	33.3	100	100	76.4
**UKELD**	**0.724**	NS	**74**	**0.298**	**100**	**38.5**	42.8	100
**APACHE 2**	0.558	NS	32	3.093	50	84.6	59.9	78.5
**APACHE 3**	0.557	NS	140	2.305	66.6	69.2	49.9	81.7
**SOFA**	0.532	NS	9	0.69	33.3	92.3	66.6	74.9
**KCH**	0.628	NS	n	0.409	33.3	92.3	66.6	74.9
**CLIF-C OF**	0.577	NS	16	0.513	100	15.4	35.3	100
**CLIF-C ACLF**	**0.660**	NS	60	0.386	100	38.5	42.8	100

AUC: area under the curve; HR, hazard ratio; PPV, positive predictive value; NPV, negative predictive value; MELD, model for end-stage liver disease; UKELD, United Kingdom model for end-stage liver disease; APACHE, acute physiology in chronic health evaluation; SOFA, Sequential organ failure assessment; CLIF-C OF, Chronic liver failure-consortium organ failures; CLIF-C ACLF, Chronic liver failure-consortium acute on chronic liver failure.

### Developing the new score

Sixty-one consecutive patients admitted from 2001 at RFH comprised the exploratory cohort. The mortality rate after 3 months from hospital admission was 37.7%. Among the predictors of mortality identified in the univariate analysis (S2 Table), GCS, mean arterial pressure, pCO2, FiO2, platelets and INR and the use of vasopressor and mechanical ventilation were excluded from the multivariate since they were part of the best three scores obtained from the previous analysis. As reported in Table 4, the following factors were significant at univariate analysis and then included in multivariate Cox regression: body temperature, heart rate, alveolar-arterial gradient, dose of Norepinephrine, platelet count, albumin, aPTT, pH, potassium, base excess, SOFA, CLIF-C OF and CLIF-C ACLF. The variables significantly associated with 3-month mortality were CLIF-C OF (p = 0.014; B = 0.391; HR = 1.478; 95%IC 1.08–2.02) and the dose of norepinephrine required to maintain mean arterial pressure >70 mmHg (p = 0.012; B = 0.020; HR = 1.021; 95%CI 1.00–1.04).

**Table 4:** Cox regression analysis of pre-transplant variables associated with 3-month mortality in not-transplanted group.

	Alive		Dead		Multivariate			
	38		23		p	B	HR	95% CI
**Body temperature**	**37.0**	**32–39**	**35.0**	**32–39**	0.539	0.131	1.14	0.75–1.73
**Heart rate (bpm)**	**105**	**32–150**	**130**	**100–160**	0.050	0.023	1.02	1.00–1.05
**A-a Gradient (mmHg)**	**39.55**	**-57–524**	**157.3**	**-79–563**	0.765	-0.01	0.99	0.99–1.00
**Norepinephrine dose (mcg/min)**	**0.00**	**0–37**	**21.33**	**0–130**	**0.012**	**0.020**	**1.02**	**1.00–1.04**
**Albumin (g/L)**	**28**	**± 6.17**	**21**	**± 4.66**	0.851	-0.01	0.99	0.91–1.08
**APTT (sec)**	**43**	**27–101**	**80**	**30–250**	0.963	0	1	0.99–1.02
**pH**	**7.387**	**± 0.96**	**7.270**	**± 0.15**	0.505	-1.57	0.21	0.00–21.2
**Potassium (mmol/L)**	**3.4**	**2.3–5.2**	**3.6**	**3.0–5.6**	0.328	-0.48	0.62	0.24–1.62
**BE (mmol/L)**	**-3**	**± 5.86**	**-9.2**	**± 6.64**	0.268	-0.04	0.95	0.87–1.04
**Lactate (mmol/L)**	**3.8**	**0.8–14.6**	**8.8**	**2.6–20.3**	0.620	-0.03	0.96	0.83–1.12
**SOFA**	**8.5**	**± 3.55**	**13.5**	**± 3.01**	0.762	-0.04	0.96	0.71–1.28
**CLIF-C OF**	**11.5**	**9–16**	**15**	**12–16**	**0.014**	**0.391**	**1.48**	**1.08–2.02**
**CLIF-C ACLF**	**45.3**	**± 14.3**	**58.7**	**± 9.3**	0.248	-0.03	0.97	0.91–1.03

The new score was calculated by multiplying the value of the predictors to their regression coefficient beta (B) and adding the results together

#### Acute liver failure-Organ failure score (ALF-OFs) = (CLIF-C OF x 0.391)+(Norepinephrine mcg/min x 0.020)

**Fig 2:**
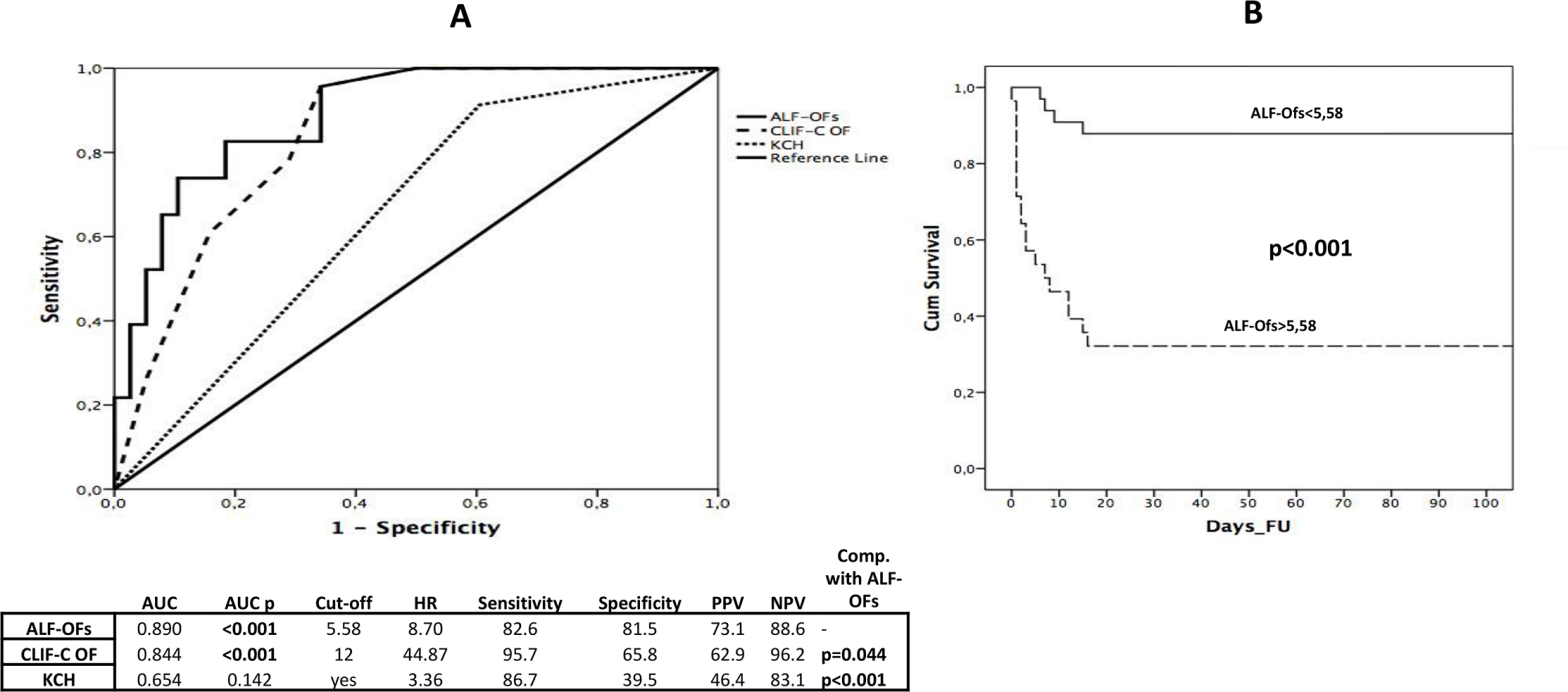
ALF OFs score characteristics. (a) ROC curves for ALF-OFs, CLIF-C OF and KCH for 3-month mortality in not-transplanted patients. (b) Kaplan Meier curve of ALF-OFs score for 3-month mortality in not-transplanted patients.

ALF-OFs was tested in the exploratory cohort and ranged between 5.2 and 11.3. In ROC analysis its AUC (0.890) was significantly higher when compared with CLIF-C OF (p = 0.044) and KCH (p<0.0001). The best cut-off was 5.58 characterized by a sensitivity of 82.6%, a negative predictive value of 89.5% and the highest specificity (89.5%) and PPV (82.6%) among the five tested scores (Fig 2A). As showed in Kaplan-Meier curve (Fig 2B), there was a significant difference in the 3-month mortality when dividing the patients according to the ALF-OFs score cut-off of 5.58 (>5.58 = 67.9% vs <5.58 = 12.1%; p<0.001). The validation cohort was composed of 26 non-transplanted patients admitted at BJH. 13/26 (50%) died within 3 months from the hospitalisation due to MOF. As showed in Fig 2B, ALF-OFs achieved an AUC of 0.988 with a sensitivity of 100% and a specificity of 92.3% at ROC analysis. Its curve was also significantly higher (p = 0.0013) when compared to KCH’s AUC (0.731).

### Risk of futile LT

We analysed 28 patients from BJH who received an emergency LT for acetaminophen-related ALF. Of them, 4 (14.3%) died within 3 days from the theatre due to a MOF and without any surgical complication. We considered them in the group that was at high risk of futility. We included in the non-futile group 24 patients that were still alive after 1 month from the liver transplant. All of the patients that died early required a multiorgan support treatment since hospital admission. They showed higher heart rate (114 vs 106bpm; p = 0.022), lower PaO_2_/FiO_2_ rates (14.9 vs 69.1kPa; p = 0.043) and required significant higher doses of Norepinephrine (68.1 vs 0 mcg/min; p = 0.035) (Table 5).

**Table 5:** Comparison between patients who died within 48 hours after LT and those that survived.

	Dead at 48 hours (n = 4)		Alive at 48 hours (n = 24)		P value
Age (years)	45.7	± 11.2	36.3	± 11.6	NS
Sex (F)	3	75%	16	66.7%	NS
Grade HE 3–4	4	100%	19	79.2%	NS
**Vasopressor use**	**4**	**100%**	**9**	**37.5%**	**0.020**
Renal replacement therapy	4	100%	13	54.2%	NS
Mechanical ventilation	4	100%	13	54.2%	NS
Body temperature (°C)	36.9	34–38.1	36.9	32.9–38.5	NS
**Heart rate (bpm)**	**114**	**± 37**	**106**	**± 21.1**	**0.022**
Mean arterial pressure (mmHg)	79	± 31.2	78	± 17.8	NS
**PaO2/FiO2 (kPa)**	**14.9**	**± 7.4**	**69.1**	**± 41.5**	**0.043**
**Norepinephrine dose (mcg/min)**	**68.1**	**33.2–265.6**	**0**	**0–132.8**	**0.035**
Ammonia (umol/L)	318	± 247	389	± 275	NS
Platelets (10^9/L)	156	± 80	131	± 78.2	NS
Creatinine (umol/L)	193	± 81	189	± 117	NS
Urea (mmol/L)	8.43	± 5.29	6.13	± 3.33	NS
Total Bilirubin (umol/L)	119	28–259	65	8–492	NS
INR	7.8	3.1–10	6.3	2.2–10	NS
pH	7.25	± 0.12	7.33	± 0.12	NS
Sodium (mmol/L)	144	138–154	137	129–148	NS
HCO3 (mmol/L)	16.5	± 6.25	15.78	± 6.22	NS
Lactate (mmol/L)	10.64	± 4.51	8.97	± 6.56	NS
**ALF-OFs score**	**8**	**6.9–12**	**5.1**	**3.5–8.5**	**0.005**

Variables are expressed as numbers and percentages or mean ± standard deviation (or median and range when appropriate). HE, hepatic encephalopathy; INR, international normalized ratio; ALF-OFs, acute liver failure-organ failures.

**Fig 3:**
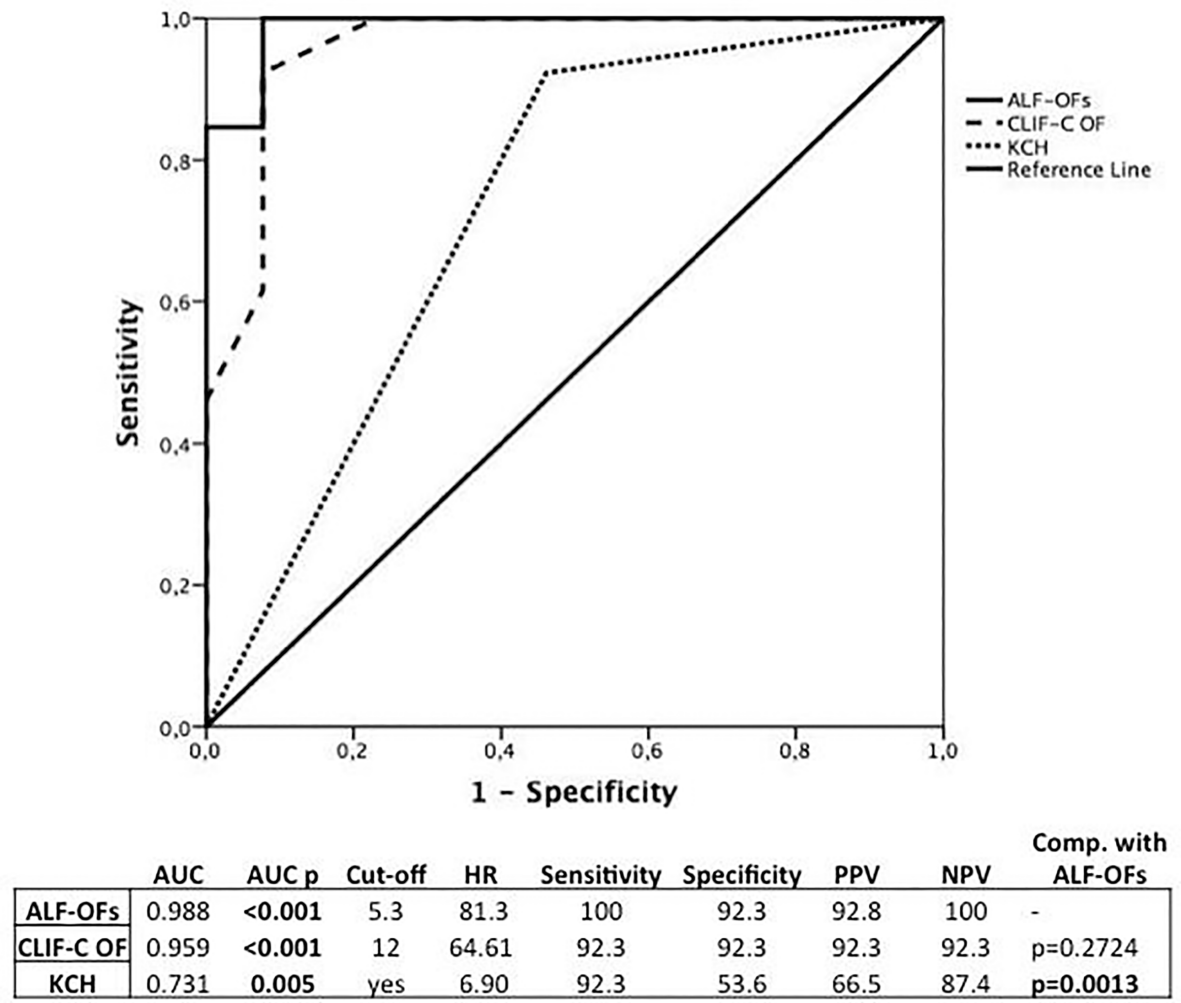
ROC curves for ALF-OFs, CLIF-C OF and KCH for patients at high risk of futility following liver transplantation.

ALF-OFs was significantly higher in those that died early on univariate Cox regression (p = 0.021, Hazard Ratio 2.955, 95% CI 1.18–7.40). At ROC analysis (Fig 3), the new score achieved an AUC of 0.917 (p<0.0001). The best cut-off was 6.5 with a 100% of sensitivity and 79.2% of specificity (PPV = 44.5%; NPV = 100%). **Fig 3**.

## Discussion

KCH criteria [5] have been extensively used to identify patients who need an emergency LT. However, recent meta-analyses showed that KCH had poor sensitivity in predicting both the LT-free mortality [6,19] and the outcome after liver transplantation [8,20]. Several attempts have been made to create new prognostic models [8,21–23]. However, most of them have underestimated the role of multiple-organ failure in the context of ALF [24]. The drastic organ shortage dictates that candidates for LT should be selected taking into account both the risk of death with medical management alone and the probability of survival after transplantation. This study aimed to develop a new prognostic score that could predict the 3-month mortality in patients with acetaminophen induced ALF and at the same time identify the patients in whom an emergency liver transplant at high risk of futility. Two cohorts from distinct intensive care units comprised our study population. They were similarly matched for age, sex, HE grade at presentation and need for organ support. The overall 3-month mortality rate in NOLT and LT cohort were similar between the two centres and in line with the currently available literature [25–27]. However, the percentage of patients who fulfilled poor prognostic criteria and were listed for LT that was higher in BJH group. It might be due to a different approach between the two centers. These potential differences may be due to the following reasons. First, in RFH group the predominant reasons for no-listing despite fulfilling KCH criteria were non-liver contraindications for liver transplantation. Second, in London the approach was to stabilize the patients before the surgery. Therefore, patients who improved with a conservative therapy were removed from the waiting list. Moreover, patients that deteriorated showing evidence of multiorgan failure and a poor response to treatment were not listed or, if listed, removed from the waiting list because the benefit of liver transplantation was considered too low. This policy could also explain the absence of futile liver transplantion in the Royal Free group. Third, the high proportion of patients considered too sick to be listed might also be due to a late referral to RFH, London. Indeed, most of them had sepsis at the admission. KCH criteria were used to select LT candidates in both cohorts. However, only one third of patients fulfilling the KCH were transplanted. Therefore, NOLT group was generated including four different categories: patients that did not fulfil KCH, those who died while on waiting list, those who improved spontaneously and those who were not listed for clinical or psychiatric reasons. This cohort was characterized by a wide range of ALF severity and it could be considered a reliable population for the creation of a new prognostic model. As the first step, we tested the performance of the main prognostic scores used in hepatology and intensive care units to predict the 3-month mortality in patients receiving LT or not. Among NOLT group, the scores that explore the severity of MOF, such as SOFA, CLIF-OF and CLIF-ACLF, showed the best AUCs. These results highlight the important prognostic role of hemodynamic dysfunction even in the early stage of ALF[24,28]. On the other hand, none of the scores calculated at admission was able to predict the 3-month mortality in LT patients, confirming that post-LT outcome is strongly affected by multiple factors including immunosuppression and graft characteristics [29]. The second step in our strategy was to look for independent predictors of 3-month transplant-free mortality. Since survival in patients with ALF has significantly improved after year 2000 due to the advances in critical medical care[29–31], we decided to analyse only patients admitted after this time point. The multivariate analysis included the scores with the best performance (SOFA, CLIF-OF and CLIF-ACLF) along with the variables tested significant in the univariate analysis and that were not present in these scores. CLIF-C OF score and the level of requirements for norepinephrine were the only significant predictors and therefore were used to build the new score. ALF-OFs score resulted a modified version of CLIF-C OF where a greater importance is given to the cardiovascular dysfunction that has been shown to significantly affect ALF patients’ outcome[24]. This could explain why our new model performs better than the existing score with a good balance between sensitivity and specificity both in the exploratory and validation cohorts.

**Fig 4:**
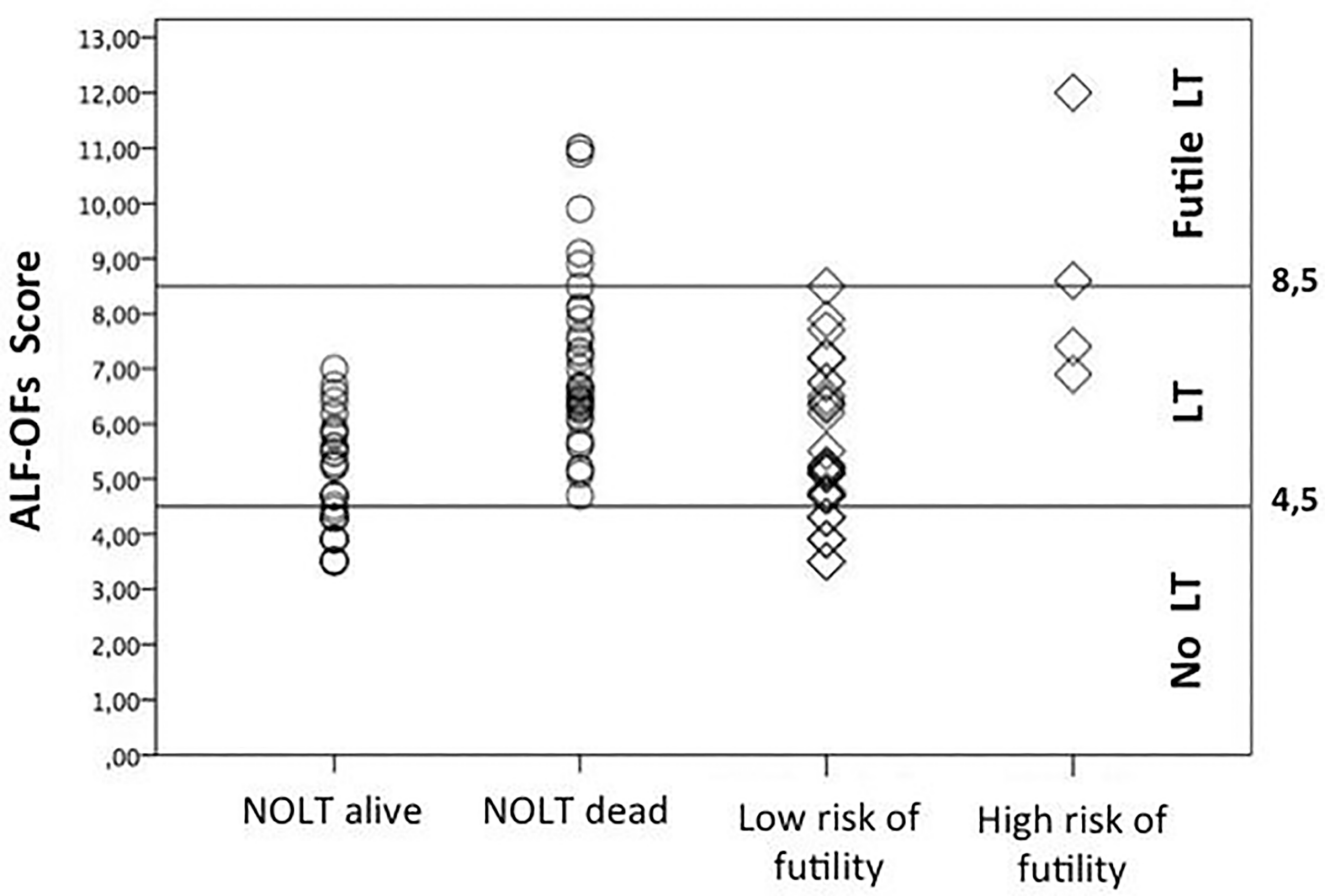
Scatter plot showing the cut-off values of ALF-OFs score for liver transplant in acetaminophen-related acute liver failure.

The last step of our study consisted of identifying the predictors of patients at high risk of early mortality after liver transplantation. Previously published studies have shown that survival after liver transplantation for ALF is still lower compared to other aetiologies, indicating that a better understanding of poor prognostic factors is mandatory to optimize organ allocation. No consensus exists on the definition of criteria that defines futility of LT[30]. The three large studies, that explored the outcome after an emergency LT, did not analyse the different aetiology (APAP-OD vs. non APAP-OD) separately and they did not discriminate patients according to cause of death [10,29,32]. We decided to focus on the highest risk group defining a high risk of futile LT as the occurrence of death within 48 hours due to MOF or irreversible brain damage that were not related to graft-function, immunosuppression and surgical complication. This definition allowed us to identify those patients in whom an emergency LT did not provide a clinical benefit. Our findings showed that early deaths after LT were characterized by a greater pre-transplant circulatory dysfunction, as suggested by the higher requirement of vasopressor in this group. ALF-OFs showed a good performance also in predicting the futility characterized by a high sensitivity (100%) associated with an acceptable specificity (79.2%). Moreover ALF-OFs allowed us to stratify the mortality risk in APAP-OD-ALF. Given the relatively small number of patients in this part of the study, we suggest caution in applying these criteria without further validation to clinical practice. In conclusion, as shown in Fig 4, we identified two different cut-offs that allowed us to further classify the patients into three categories; patients who are likely to survive without a LT (ALF-OFs<4.5); patients with a high risk to die without a LT (ALF-OFs 4.5–8.5); and those where a LT is at high risk of being futile (ALF-OFs>8.5). We acknowledge that the numbers of patients are limited and larger studies are needed to further investigate this topic. However, this study points to the role of multiple organs in defining the outcome of ALF patients and describes a new prognostic score based on pre-LT variables to define the need for LT and early post LT mortality.

## Supporting information

S1 Table Clinical and laboratory characteristics of patients. (PDF)

S2 Table Univariate and multivariate analysis. (PDF)

## Abbreviations

A-a gradient: Alveolar-arterial gradient
ALF: acute liver failure
ALF-OFs: acute liver failure-organ failures
APACHE: acute physiology in chronic health evaluation
APTT: activated partial thromboplastin time
AUC: area under the curve
BE: base excess
BJH: Beaujon hospital
CLIF-C ACLF: Chronic liver failure-consortium acute on chronic liver failure
CLIF-C OFs: Chronic liver failure-consortium organ failures
FiO_2_: fraction of inspired oxygen
GCS: Glasgow coma scale
HE: hepatic encephalopathy
HR: hazard ratio
INR: international normalized ratio
KCH: King’s college hospital
LT: liver transplantation
MELD: model for end-stage liver disease
MOF: multi-organ failure
NOLT: not-transplanted
NPV: negative predictive value
NS: not significant
PaO_2_: partial pressure of arterial oxygen
PaCO_2_: partial pressure of carbon dioxide
APAP-OD: acetaminophen overdose
PPV: positive predictive value
RFH: Royal Free Hospital
SOFA: Sequential organ failure assessment
UKELD: United Kingdom model for end-stage liver disease
