# Supplementary material for: Development of an organ failure score in acute liver failure for transplant selection and identification of patients at high risk of futility
Source: PLoS One. 2017 Dec 5;12(12):e0188151. doi: 10.1371/journal.pone.0188151 (PMC5716582; doi:10.1371/journal.pone.0188151)
Supplement: S2 Table — (PDF) [file pone.0188151.s002.pdf]

	**Alive**		**Dead**		**Univariate**			**Included in SOFA, CLIF-OF or CLIF-ACLF**	**Multivariate**			
	**38**		**23**		**P value.**	**HR**	**95% CI**		**p**	**B**	**HR**	**95% CI**
Age (years)	37.9	± 11.5	42.9	± 15.5	0.184	1.020	0.99 - 1.05	yes				
Sex (F)	25	65.8%	12	52.2%	0.228	0.604	0.27 - 1.37	no				
Grade HE 3-4	26	68.4%	12	52.2%	0.221	0.599	0.26 - 1.36	yes				
**Vasopressor use**	**12**	**31.6%**	**21**	**91.3%**	**<0.001**	**13.322**	**3.11 - 57.1**	yes				
Renal replacement therapy	19	50%	16	69.6%	0.211	1.764	0.73 - 4.29	yes				
**Mechanical ventilation**	**18**	**47.4%**	**21**	**91.3%**	**0.005**	**8.037**	**1.88 - 34.4**	yes				
**GCS**	**13**	**3-15**	**8**	**3-15**	**0.038**	**0.914**	**0.84 - 0.99**	yes				
**Body temperature (°C)**	**37.0**	**32.2 - 39.2**	**35.0**	**32.6 - 39.1**	**0.023**	**0.762**	**0.60 - 0.96**	**no**	0.539	0.131	1.14	0.75 - 1.73
**Heart rate (bpm)**	**105**	**32 - 150**	**130**	**100 - 160**	**0.001**	**1.041**	**1.02 - 1.07**	**no**	0.050	0.023	1.023	1.00 - 1.05
**Mean arterial pressure (mmHg)**	**72**	**± 11.2**	**53**	**± 11.2**	**<0.001**	**0.928**	**0.89 - 0.96**	yes				
Respiratory rate (bpm)	20	10-46	22	12-46	0.332	1.023	0.98 - 1.07	no				
**PaCO2 (kPa)**	**4.32**	**± 0.95**	**5.01**	**± 1.45**	**0.017**	**1.436**	**1.07 - 1.93**	yes				
PaO2 (kPa)	12.97	6.3 - 36.5	14.57	14.6 - 48.7	0.133	1.047	0.99 - 1.11	yes				
**FiO2 (%)**	**28**	**21 - 100**	**40**	**28 - 100**	**0.002**	**1.023**	**1.00 - 1.04**	yes				
PaO2/FiO2 (kPa)	53	± 19.6	34	± 24.1	0.070	0.98	0.96 - 1.00	yes				
**A-a Gradient (mmHg)**	**39.55**	**-581**	**157.3**	**-642**	**0.008**	**1.003**	**1.00 - 1.01**	**no**	0.765	-0.001	0.999	0.99 - 1.00
**Norepinephrine dose (mcg/min)**	**0.00**	**0 - 37**	**21.33**	**0 - 130**	**<0.001**	**1.038**	**1.03 - 1.05**	**no**	**0.012**	**0.020**	**1.021**	**1.00 - 1.04**
Ammonia (umol/L)	82	± 49.8	119	± 61.6	0.219	1.009	0.99 - 1.02	no				
White blood cells (10^9/L)	9.3	± 6.2	10.5	± 6.8	0.388	1.028	0.97 - 1.09	yes				
**Platelets (10^9/L)**	**109**	**± 65.8**	**48**	**± 53.0**	**0.019**	**0.99**	**0.98 - 0.99**	yes				
Hematocrit (L/L)	0.284	± 0.06	0.261	± 0.07	0.201	0.011	0.00 - 11.1	no				
Creatinine (umol/L)	157	43 - 825	204	85 - 663	0.809	1	0.99 - 1.00	yes				
Urea (mmol/L)	8	1.9 - 46.9	7.3	3.7 - 28.2	0.744	0.991	0.94 - 1.04	yes				
Total Bilirubin (umol/L)	70	3 - 581	93	16 - 414	0.812	1	0.99 - 1.00	yes				
**Albumin (g/L)**	**28**	**± 6.17**	**21**	**± 4.66**	**<0.001**	**0.885**	**0.83 - 0.94**	**no**	0.851	-0.008	0.992	0.91 - 1.08
C reactive protein (mg/L)	10.5	1-67	6	1 - 102	0.936	0.999	0.97 - 1.02	no				
**INR**	**5.7**	**1.2 - 8**	**8**	**1.8 - 15**	**0.001**	**1.316**	**1.11 - 1.55**	yes				
**APTT (sec)**	**43**	**27 - 101**	**80**	**30 - 250**	**<0.001**	**1.015**	**1.01 - 1.02**	**no**	0.963	0	1	0.99 - 1.02
**pH**	**7.387**	**± 0.96**	**7.270**	**± 0.15**	**<0.001**	**0.001**	**0.00 - 0.03**	**no**	0.505	-1.576	0.207	0.00 - 21.2
Sodium (mmol/L)	131	± 7.18	126	± 9.38	0.280	0.972	0.92 - 1.02	no				
**Potassium (mmol/L)**	**3.4**	**2.3 - 5.2**	**3.6**	**3.0 - 5.6**	**0.015**	**2.408**	**1.18 - 4.90**	**no**	0.328	-0.48	0.619	0.24 - 1.62
HCO3 (mmol/L)	20.3	± 4.81	18.0	± 5.11	0.064	0.921	0.85 - 1.01	no				
**BE (mmol/L)**	**-3**	**± 5.86**	**-9.2**	**± 6.64**	**0.004**	**0.91**	**0.85 - 0.97**	**no**	0.268	-0.049	0.952	0.87 - 1.04
**Lactate (mmol/L)**	**3.8**	**0.8 - 14.6**	**8.8**	**2.6 - 20.3**	**<0.001**	**1.175**	**1.09 - 1.26**	**no**	0.620	-0.037	0.963	0.83 - 1.12
**SOFA**	**8.5**	**± 3.55**	**13.5**	**± 3.01**	**<0.001**	**1.313**	**1.17 - 1.48**	**-**	0.762	-0.045	0.956	0.71 - 1.28
**CLIF-C OF**	**11.5**	**9-16**	**15**	**12-16**	**<0.001**	**1.705**	**1.31 - 2.22**	**-**	**0.014**	**0.391**	**1.478**	**1.08 - 2.02**
**CLIF-C ACLF**	**45.3**	**± 14.3**	**58.7**	**± 9.3**	**<0.001**	**1.079**	**1.03 - 1.13**	**-**	0.248	-0.035	0.965	0.91 - 1.03
