# Supplementary material for: Development of an organ failure score in acute liver failure for transplant selection and identification of patients at high risk of futility
Source: PLoS One. 2017 Dec 5;12(12):e0188151. doi: 10.1371/journal.pone.0188151 (PMC5716582; doi:10.1371/journal.pone.0188151)
Supplement: S1 Table — (PDF) [file pone.0188151.s001.pdf]

Patient code	Center	Age	Sex	3-month outcome	Death post liver transplantation	Cause of death	Futile liver transplant	King's College criteria	Listed	Cause of no listing	Death before Tx	Reason no transplant despite listed	Liver transplant	Sepsi
P0067	RFH	38	M	death		MOF		y	y		y	died in list	no	no
P0024	RFH	36	F	death		MOF		y	y		y	died in list	no	no
P0124	RFH	33	M	death		MOF		y	y		y	died in list	no	no
P0156	RFH	38	F	alive	yes		No Futile	y	y		n		yes	no
P0055	RFH	50	F	death		MOF		y	y		y	died in list	no	yes
P0078	RFH	63	F	death		Sepsis		y	n	Clinical			no	yes
P0092	RFH	29	M	alive	no		No Futile	y	y		n		yes	no
P0061	RFH	36	F	alive				y	n	improved			no	yes
P0152	RFH	35	F	alive				y	n	improved			no	no
P0072	RFH	30	F	alive	yes		No Futile	y	y		n		yes	no
P0046	RFH	17	F	alive	yes		No Futile	y	y		n		yes	no
P0178	RFH	21	F	death	yes	Rise of intracranial pressure due a mid brain herniation refractary to therapy. Go	Futile	y	y		n		yes	no
P0115	RFH	25	F	death		MOF		y	y		y	died in list	no	yes
P0138	RFH	79	F	death		MOF		y	n	Psychiatric			no	yes
P0136	RFH	36	M	death		MOF		y	n	Psychiatric			no	no
P0129	RFH	32	M	death		MOF		y	y		y	died in list	no	no
P0071	RFH	35	M	alive				y	n	improved			no	yes
P0113	RFH	47	M	death		MOF		y	y		y	died in list	no	no
P0154	RFH	43	F	alive				y	n	improved			no	no
P0120	RFH	46	M	death		MOF		y	n	Clinical			no	no
P0128	RFH	47	F	death		Sepsis		y	n	Clinical			no	yes
P0062	RFH	41	F	alive				y	n	improved			no	no
P0109	RFH	70	M	death		MOF		y	n	Clinical			no	yes
P0103	RFH	39	F	alive	no		No Futile	y	y		n		yes	yes
P0082	RFH	43	M	death		MOF		y	n	Psychiatric			no	no
P0191	RFH	33	F	death	yes	Sepsis	No Futile	y	y		n		yes	no
P0166	RFH	53	F	death		Sepsis		y	n	Clinical			no	yes
P0133	RFH	46	M	death		MOF		y	n	Clinical			no	no
P0006	RFH	36	F	alive				y	n	improved			no	no
P0118	RFH	42	F	death		MOF		y	n	Clinical			no	no
P0027	RFH	57	F	death		Sepsis		y	n	Clinical			no	yes
P0104	RFH	38	F	alive				y	y		n	improved	no	yes
P0169	RFH	49	F	alive	no		No Futile	y	y		n		yes	no
P0194	RFH	34	M	death		MOF		y	n	Clinical			no	no
P0117	RFH	49	M	alive				y	n	improved			no	no
P0097	RFH	40	M	alive				y	y		n	improved	no	no
P0163	RFH	31	F	death		MOF		y	y		y	died in list	no	no
P0183	RFH	26	F	alive	no		No Futile	y	y		n		yes	no
P0116	RFH	35	F	death		MOF		y	n	Psychiatric			no	no
P0110	RFH	19	F	death		MOF		y	n	Clinical			no	no
P0019	RFH	33	M	alive				y	n	improved			no	no
P0059	RFH	59	M	alive				y	y		n	improved	no	yes
P0200	RFH	20	F	death	yes	Sepsis	Futile	y	y		n		yes	no
P0028	RFH	43	M	alive				y	n	improved			no	no
P0162	RFH	63	M	alive				y	n	Psychiatric			no	no
P0167	RFH	60	M	alive				n	n	no fulfil criteria			no	no
P0197	RFH	50	F	death		MOF		y	n	Clinical			no	yes
P0157	RFH	30	F	alive				y	y		n	improved	no	yes
P0143	RFH	37	F	alive				y	n	improved			no	yes
P0083	RFH	52	F	alive	no		No Futile	y	y		n		yes	no
P0123	RFH	47	F	alive				y	n	improved			no	no
P0085	RFH	43	M	alive				n	n	no fulfil criteria			no	no
P0029	RFH	21	F	death		MOF		y	n	Clinical			no	no
P0146	RFH	19	F	death	yes	Sepsi	No Futile	y	y		n		yes	no
P0089	RFH	24	M	death		Severe brain injuri		y	n	Psychiatric			no	no
P0170	RFH	26	F	alive	no		No Futile	y	y		n		yes	no
P0094	RFH	20	F	alive	no		No Futile	y	y		n		yes	no
P0159	RFH	64	F	death		MOF		y	n	Psychiatric			no	yes
P0192	RFH	15	F	alive				y	y		n	improved	no	no
P0056	RFH	63	M	death		MOF		y	n	Psychiatric			no	yes
P0172	RFH	27	M	death		MOF		y	n	Clinical			no	no
P0181	RFH	27	F	alive				y	n	improved			no	no
P0190	RFH	28	F	death		Sepsis		y	n	Clinical			no	yes
P0141	RFH	46	F	alive	no		No Futile	y	y		n		yes	no
P0100	RFH	24	F	death	yes	Sepsis	No Futile	y	y		n		yes	yes
P0142	RFH	41	M	alive	yes		No Futile	y	y		n		yes	no
P0068	RFH	26	F	alive				n	n	no fulfil criteria			no	yes
P0135	RFH	44	F	alive				n	n	no fulfil criteria			no	no
P0177	RFH	23	F	alive				n	n	no fulfil criteria			no	no
P0026	RFH	57	M	alive				y	n	improved			no	no
P0132	RFH	26	F	alive				y	n	improved			no	yes
P0160	RFH	28	M	death		Sepsis		y	n	Clinical			no	yes
P0121	RFH	37	F	death		Sepsis		y	n	Clinical			no	yes
P0106	RFH	29	F	alive				y	n	improved			no	yes
P0130	RFH	51	M	alive				y	n	improved			no	no
P0030	RFH	56	F	alive				y	n	improved			no	yes
P0122	RFH	29	F	alive				y	n	improved			no	no
P0147	RFH	25	M	death	yes	Sepsi	No Futile	y	y		n		yes	no
P0140	RFH	44	F	alive				y	n	improved			no	no
P0093	RFH	36	F	alive				n	n	no fulfil criteria			no	no
P0095	RFH	20	M	alive				n	n	no fulfil criteria			no	no
P0137	RFH	20	M	alive				n	n	no fulfil criteria			no	no
P0070	RFH	32	F	alive				n	n	no fulfil criteria			no	no
P0090	RFH	57	F	alive				y	n	Psychiatric			no	yes
P0125	RFH	33	F	alive				y	n	Psychiatric			no	no
P0076	RFH	44	F	death	yes	Suicide	No Futile	y	y		n		yes	no
P0080	RFH	25	M	alive				n	n	no fulfil criteria			no	no
P0112	RFH	36	M	alive				n	n	no fulfil criteria			no	no
P0074	RFH	35	F	alive				n	n	no fulfil criteria			no	yes
P0155	RFH	31	M	alive				n	n	no fulfil criteria			no	no
P0038	RFH	31	F	alive				y	n	improved			no	no
P0114	RFH	69	F	alive				y	n	improved			no	yes
P0009	RFH	34	F	alive				y	n	improved			no	no
P0034	RFH	39	F	alive				n	n	no fulfil criteria			no	no
P0158	RFH	25	F	alive				n	n	no fulfil criteria			no	no
P0048	RFH	27	F	alive				y	n	improved			no	no
P0011	RFH	25	F	alive				y	n	improved			no	no
P0037	RFH	26	F	alive				y	n	improved			no	no
F025	BJ	44	M	death	yes	MOF	Futile	y	y				yes	
F019	BJ	61	M	death		MOF		y	y				no	
F058	BJ	52	M	death		Cardiac arrest at admission (resuscitated) a second one7 hours after admission		y	n	Clinical			no	
F012	BJ	29	F	death		MOF		y	y				no	
F008	BJ	59	F	death		MOF		y	y				no	
F059	BJ	45	F	death		Cardiac arrest on WL while under NAD 11mg/h		y	y				no	
F038	BJ	57	M	death		Died at 6mg/h		y	y				no	
F021	BJ	57	F	death	yes	ARDS and MOF 24h after LT	Futile	y	y				yes	
F003	BJ	44	F	alive	no		No Futile	y	y				yes	
F035	BJ	46	M	death		Decision not to perform LT aftes hepatectomy (NAD 18mg/h and AD 8mg/h)		y	y				no	
F033	BJ	38	F	alive	no		No Futile	y	y				yes	
F030	BJ	43	M	death		Not listed due to social problems		n	n				no	
F053	BJ	51	F	alive	no		No Futile	y	y				yes	
F011	BJ	58	M	death		Decision not to perform LT aftes hepatectomy (NAD 20mg/h and AD 8mg/h)		y	y				no	
F002	BJ	51	F	death	yes	Cardiac arrest during hepatectomy	Futile	y	y				yes	
F028	BJ	29	M	death		Deceased in the OR during hepatectomy (cardiac arrest under NAD 5mg/h)		y	y				no	
F047	BJ	37	M	alive	no		No Futile	y	y				yes	
F044	BJ	17	F	alive	no		No Futile	y	y				yes	
F068	BJ	60	F	death		Died frompneumocoque meningitis, ALF resolved		y	n	Clinical			no	
F018	BJ	31	F	death	yes	Cardiac arrest due to hypoxemia at D3 of admission, D0 of LT, ARDS criteria just a	Futile	y	y				yes	
F026	BJ	41	M	alive				y	n	improved			no	
F014	BJ	24	F	alive	no		No Futile	y	y				yes	
F005	BJ	53	F	death		MOF and cardiac arrest after induction, ischemic bowel, decision not to perform LT		y	y				no	
F050	BJ	45	F	death	yes	Deceased at D51 after necrotizing fasciitis on pucture site / good liver function	No Futile	y	y				yes	
F042	BJ	53	M	alive	no		No Futile	y	y				yes	
F037	BJ	19	F	death		Died from intracranial hypertension		y	y				no	
F066	BJ	28	F	alive	no		No Futile	y	y				yes	
F010	BJ	50	F	alive				n	n				no	
F015	BJ	48	M	alive	no		No Futile	y	y				yes	
F036	BJ	40	F	alive	no		No Futile	y	y				yes	
F070	BJ	34	F	alive	no		No Futile	y	y				yes	
F048	BJ	22	F	alive	no		No Futile	n	y				yes	
F049	BJ	59	F	alive	no		No Futile	y	y				yes	
F001	BJ	45	M	alive	no		No Futile	y	y				yes	
F020	BJ	48	F	alive	no		No Futile	y	y				yes	
F041	BJ	42	F	alive	no		No Futile	n	y				yes	
F039	BJ	45	M	alive				y	y				no	
F063	BJ	37	M	alive	no		No Futile	y	y				yes	
F029	BJ	18	M	alive	no		No Futile	y	y				yes	
F013	BJ	37	F	alive				y	n	improved			no	
F061	BJ	31	F	alive				y	y				no	
F064	BJ	21	F	alive				y	y				no	
F007	BJ	44	M	alive				n	y				no	
F024	BJ	24	M	alive	no		No Futile	y	y				yes	
F054	BJ	22	F	alive	no		No Futile	y	y				yes	
F055	BJ	35	M	alive	no		No Futile	n	y				yes	
F043	BJ	60	F	alive				n	n				no	
F032	BJ	21	F	alive				y	y				no	
F067	BJ	17	F	alive				n	n				no	
F023	BJ	34	F	alive	no		No Futile	y	y				yes	
F060	BJ	28	F	alive	no		No Futile	y	y				yes	
F009	BJ	49	F	alive				n	n				no	
F006	BJ	20	F	alive				n	y				no	
F017	BJ	19	F	alive				n	y				no	

Patient code	Ammonia (umol/L)	Grade of Haepatic Enchephalopathy	Glasgow Coma Scale	Body temperature (°C)	Heart rate (bpm)	Mean arterial pressure (mmHg)	Respiratory rate (bpm)	White blood cell count (10^9/L))	Platelets count (10^9/L)	Hematocrit (L/L)	Creatinine (umol/L)	Urea (mmol/L)	Total Bilirubin (umol/L)	Albumine (g/L)	C reactive proteine (mg/L)	PT-INR	APTT (sec)	pH	Sodium (mmol/L)	Potassium (mmol/L)
P0067	140	3-4	3	35,4	135	52	22	14,9	19	0,263	204	7,1	51	16	6	8	101	7,182	135	4
P0024		3-4	9	37,5	140	57	22	16,6	93	0,376	140	12,2	93	32	1	8	57	6,994	120	5,6
P0124		<3	3	34	150	50	16	24,7	8	0,375	230	6,5	63	20	3	10,7	134	6,94	122	3,9
P0156		<3	8	34,5	125	48	16	9,8	25	0,18	335	4,7	73			8	250	7,27	121	3,6
P0055	91	3-4	13	34,7	120	52	36	20,9	112	0,229	114	4,1	44	21	5	8	59	7,465	128	3,3
P0078		<3	3	34,8	130	43	30	9,4	28	0,218	184	6,4	69	17	6	8	90	7,2	134	4
P0092	286	3-4	5	32,6	110	60	16	0,8	52	0,234	693	11,9	187	26	10	7,4	85	7,3	121	5,4
P0061	109	<3	3	35	120	55	18	4,5	116	0,239	91	7,3	40		5	8	43	7,11	130	3,9
P0152		3-4	3	38	165	58	12	8,6	14	0,29	265	8,4	186	21		4,3	109	7,18	133	3,4
P0072	69	<3	3	37	130	60	18	11,4	45	0,213	278	3,9	88	16	8	7,4	72	7,421	142	3,2
P0046	127	3-4	12	34,7	110	63	38	21	48	0,178	297	8,3	133	20	2	8	61	7,21	133	3,8
P0178		3-4	3	36,5	140	43	12	22,1	81	0,26	247	5,2	75	38		4	59	7,05	134	3,9
P0115		3-4	9	34,9	125	48	26	10,5	48	0,258	320	4,8	414	22	1	8	180	7,28	123	3,8
P0138		<3	3	34	100	50	18	9	41	0,192	200	15,4	16	15	14	10	88	7,16	132	4,1
P0136		3-4	3	37,2	125	48	14	2,3	33	0,164	663	28,2	114	16	7	10,8	54	7,42	139	3,3
P0129		3-4	12	35,5	140	47	18	11,1	26	0,32	133	6,2	69	29	6	8	142	7,27	126	3,5
P0071	52	<3	3	37	150	60	21	21,2	52	0,22	210	9,7	45	23	62	6	61	7,21	134	5,2
P0113		<3	8	34,4	120	72	15	15,9	61	0,206	140	4,1	45	14	2	8	138	7,21	135	3,4
P0154		3-4	3	35	130	44	16	14,4	63	0,32	155	10,7	88	28		8	100	7,216	131	3,2
P0120		3-4	9	32,6	115	53	12	19,3	39	0,162	285	10,9	55	21		8	150	7	127	3,5
P0128		<3	3	36,5	130	70	17	19,6	211	0,313	427	21,5	37	27	36	5,3	47	7,229	121	3,5
P0062	124	3-4	10	34	90	77	18	10	102	0,299	483	19,8	154	27	11	3	41	7,33	127	3,4
P0109		3-4	15	34,7	130	43	20	3,4	34	0,171	242	7,3	93	21	6	2,8	77	7,251	121	3,8
P0103		<3	13	34,3	120	60	14	16,4	116	0,209	368	13,2	104	22	3	8	60	7,21	123	2,7
P0082	219	3-4	15	37,1	110	47	30	2	86	0,235	174	12	99	21	35	8	89	7,319	124	4,1
P0191		<3	5	37,5	140	62	36	9,9	27		508	4,9	318	38		8	95	7,21	134	3,2
P0166		<3	8	35	130	78	15	13,7	66	0,35	290	12	115	41		13,7	61	7,34	125	3,6
P0133		<3	14	35	160	57	46	0,6	42	0,28	293	11,3	50	17		15	58	7,1	124	4
P0006	68	3-4	11	35	55	78	22	13,9	46	0,233	261	7,8	93	30	1	7,3	43	7,2	134	3,5
P0118		<3	10	34	135	43	42	6,6	70	0,215	309	5,8	164	23	8	4,7	80	7,42	129	3
P0027	48	<3	8	39,1	115	67	26	15,3	110	0,41	85	7	216	24	102	2,9	53	7,35	150	3,8
P0104		3-4	10	34,1	45	67	35	4,6	38	0,27	328	15,8	143	28	13	8	62	7,32	133	2,8
P0169		3-4	3	35	110	62	12	13,8	147	0,27	243	6,6	50	31		7	74	7,378	132	3,9
P0194		3-4	3	38	150	93	12	12,6	28		139	4,8	157	36		6	62	7,45	132	3,9
P0117		3-4	14	35,8	130	63	41	1,7	56	0,217	315	16	129	20	23	8	82	7,27	130	4
P0097		3-4	14	35	130	63	46	11,2	139	0,202	308	6,7	56	9	17	8	101	7,367	123	3
P0163		3-4	7	34,5	115	80	24	11,1	116	0,26	277	13,9	73	31		16	84	7,4	129	3,9
P0183		3-4	9	37	160	50	28	24,8	52	0,25	272	8,6	68	30		8	100	7,25	127	2,8
P0116		3-4	8	37,3	150	53	23	12,8	113	0,323	168	5,8	116	19	4	8	250	7,22	122	3,1
P0110		<3	12	36,3	110	63	21	8,4	148	0,26	254	14	99	25	32	6,8	48	7,427	122	3,4
P0019	97	<3	13	34	32	80	14	25,5	94	0,351	97	6,3	38	38	6	3,8	35	7,31	136	3,2
P0059	206	<3	3	34,5	115	62	14	13,7	106	0,263	249	8,2	79	21	4	8	62	7,335	130	3,1
P0200		3-4	3	34	130	48	15	19,8	134		210		96	43		6	45	7,26	142	4,6
P0028	136	3-4	9	36,4	85	57	14	4,6	59	0,254	470	15,1	150	24	22	3,3	40	7,307	122	3,5
P0162		3-4	3	31	125	73	12	3,4	61	0,24	187	16,4	77	36		8	41	7,43	127	3
P0167		3-4	15	37	80	77	22	13,3	44	0,35	319	14,6	171	41		7,5	73	7,426	135	3,8
P0197		<3	3	38	140	63	43	9	72		439	9,7	89	35		8	79	7,31	123	3,1
P0157		3-4	3	36	135	58	14	19,8	89		90	3,1	25	33		1,9	58	7,29	131	3,5
P0143		3-4	13	36,6	100	70	14	19,8	80	0,263	488	14,5	75	33	18	8	63	7,3	120	3,3
P0083	130	<3	3	34,8	120	60	20	15	31	0,229	253	8,7	73	23	1	8	143	7,27	130	3,3
P0123		<3	14	37,6	130	53	27	16,9	95	0,19	118	6,9	45	27	9	8	78	7,46	123	3,5
P0085	35	3-4	3	39,2	80	63	15	6,6	113	0,29	46	2,3	53	22	31	2,7	40	7,43	131	3
P0029		3-4	3	34,2	150	75	22	5,9	63	0,191	117	7,6	54	27	1	1,8	30	7,36	137	3,5
P0146		3-4	6	35	160	60	56	20,8	99	0,322	110	2,4	113	35		8	107	7,047	133	3,6
P0089	171	<3	6	34	100	53	14	3,9	37	0,34	105	3,7	206	23	4	8	44	7,46	147	3,2
P0170		3-4	3	34	140	62	27	15,9	117	0,4	98	3,9	128	42		8	114	7,416	135	3,5
P0094	246	<3	13	34,3	140	83	30	8,8	78	0,2	466	8,9	117	16	5	6,6	58	7,22	123	3,6
P0159		<3	8	37	105	77	16	1,4	27	0,18	544	21,1	145	28		2,9	40	7,33	122	4,1
P0192		3-4	14	37	120	97	23	9	122		348	11,2	86	42		8	23	7,387	122	3,3
P0056	98	<3	8	36,3	120	72	24	7	180	0,296	257	8,6	114	18	32	4,4	67	7,282	112	3,6
P0172		<3	15	37	140	60	24	20,2	21	0,31	275	9,9	46	35		8	180	7,291	129	4,6
P0181		<3	13	35,5	160	57	28	5,5	69	0,33	397	6,3	121	41		5,6	50	6,93	132	4,5
P0190		3-4	15	37,5	120	73	28	22,1	60		417	11,4	99	36		8	75	7,29	136	4,6
P0141		3-4	3	37	130	80	12	16,1	239	0,279	107	7,8	43	17	5	15,3	96	7,4	125	3,4
P0100		<3	13	35	120	83	21	17,1	73	0,309	217	3,2	115	25	4	8	118	7,46	131	3,4
P0142		3-4	12	35,5	145	90	22	16	44	0,327	317	17,8	76	45	8	8	180	7,4	126	3,6
P0068	85	3-4	3	37,5	90	63	14	2,6	66	0,349	43	2,8	58	26	6	3,7	42	7,37	141	3,1
P0135		3-4	13	38	140	77	29	2,7	29	0,195	297	21,9	73	30	5	4,8	56	7,394	120	4,3
P0177		3-4	10	39	90	65	24	15,2	159	0,3	73	1,8	90	43		8	75	7,471	133	3,3
P0026		<3	14	38	120	85	26	5,3	6	0,288	413	11,6	141	34	8	3,7	46	7,425	131	3,4
P0132		<3	14	37,5	130	70	22	18,3	66	0,258	314	7,3	115	21	6	8	98	7,44	117	3,3
P0160		3-4	12	35,5	135	47	25	43,5	46	0,12	223	13,7	55	17		3	60	7,35	129	4,4
P0121		3-4	14	37	130	80	26	18,2	43	0,2	269	13,9	149	24	5	8	110	7,33	116	3,8
P0106		3-4	15	37,7	120	80	24	3,1	84	0,245	366	10,6	154	23	22	4,3	41	7,38	131	3,5
P0130		3-4	14	37,2	100	83	25	3,5	29	0,34	345	10,9	137	29	14	6,4	73	7,48	122	3,8
P0030		3-4	10	32,2	140	58	14	17	197	0,363	61	6,7	3	42	1	1,4	32	7,149	137	3,3
P0122		<3	15	38,2	120	70	32	6,8	288	0,237	121	3,4	34	19	27	5,8	82	7,479	131	3,7
P0147		3-4	3	35	100	63	16	23,7	130	0,31	107	5,3	84	20		14,7	85	7,39	136	4,5
P0140		3-4	3	35	70	82	12	3,1	153	0,22	501	41,1	41	29	27	2,2	32	7,36	112	4,1
P0093		3-4	14	36,1	112	80	10	11,7	132	0,31	825	46,9	67	30	11	3,5	41	7,45	122	2,8
P0095	184	3-4	11	38	45	83	10	11,3	116	0,218	108	6,6	126	26	8	3,6	43	7,49	140	3,3
P0137		3-4	14	37	115	87	25	6	89	0,35	183	11,2	102	35	10	4,4	43	7,515	131	3,1
P0070	78	3-4	12	37,5	100	70	18	20,6	235	0,195	62	9,3	581	34	23	8	47	7,41	139	3,9
P0090		3-4	13	38	120	93	32	1,6	10	0,357	124	12,1	46	24	67	5,6	55	7,311	141	3,4
P0125		3-4	15	37	120	73	24	10,8	205	0,254	114	5,9	52	27		8	63	7,45	131	3,5
P0076		3-4	13	37,4	130	75	23	15,9	170	0,327	46	2,8	101	20	1	8	180	7,34	136	3,3
P0080	62	<3	3	38	90	83	16	8,3	133	0,417	152	12,7	204	30	17	2,1	34	7,35	141	3,8
P0112		<3	15	36,8	55	83	18	8,5	150	0,392	161	4,4	106	32	4	8	59	7,47	124	3,3
P0074		3-4	14	37,7	70	80	23	5,6	122	0,318	273	9	40	31	17	3,3	34	7,44	131	2,3
P0155		3-4	15	38	100	77	25	10,4	114	0,39	118	8	144	33		6	46	7,4	138	3,2
P0038	58	<3	15	37,1	120	77	20	7	141	0,37	68	10	114	31	10	6,1	31	7,44	128	3,4
P0114		3-4	6	32,9	75	47	12	3,7	176	0,308	90	5,1	13	22		1,9	42	7,32	129	3,2
P0009	76	3-4	15	38,2	132	63	19	14	120	0,322	47	2,3	90	33	1	8	34	7,46	139	3
P0034		<3	5	36,5	110	63	16	12,3	252	0,287	53	1,9	5	29	5	1,2	27	7,376	139	3,2
P0158		3-4	15	37	100	87	24	7,3	114	0,4	121	6,7	72	31		3,7	42	7,437	134	3,5
P0048		3-4	15	37	75	83	20	13,4	123	0,345	53	4,8	46	25	26	5,5	40	7,398	135	3,6
P0011		3-4	14	37,5	95	87	20	11,9	193	0,288	62	6	50	31	4	8	32	7,49	134	3
P0037		3-4	13	37,5	100	70	23	10,1	102	0,29	47	4	58	28	3	8	36	7,42	133	3,7
F025	672	3-4		38,1	144	69			70		295	16,1	162			9,3		7,25	149	
F019	173	3-4		36,5	112	63			335		564	17,8	248			11,9		7,48	129	
F058	690	3-4		37	120	47			155		558	11,4	106			9,3		6,92	133	
F012	360	3-4		36,1	133	65			9		70	2,6	80			10,7		7,03	146	
F008	309	3-4		35,7	98	80			155		153	3,3	59			7,7		7,45	139	
F059	109	<3		37,2	105	63			461		150	2,3	46			5		7,25	140	
F038	767	3-4		37,3	110	40			33		388	14,5	31			10		6,92	143	
F021	221	3-4		34	88	73			156		125	4	259			10		7,14	154	
F003	364	3-4		38,4	120	63			127		299	6,2	67			3,1		7,22	147	
F035	748	3-4		36	100	47			45		351	9,5	62			7,6		7,26	127	
F033	284	3-4		38,5	133	84			121		90	4,2	35			7,3		7,35	129	
F030	158	<3		35,8	87	89			129		274	10,5	486			11,9		7,42	130	
F053	62	3-4		36,9	100	75			121		341	16,2	15			2,2		7,34	145	
F011	999	3-4		34,2	93	68			10		238	13,7	60			5,4		7,13	142	
F002	281	3-4		35,9	76	50			262		132	6,7	76			6,3		7,42	138	
F028	686	3-4		36	112	56			209		206	5,7	73			9,3		7,09	131	
F047		3-4		32,9	83	35			90		164	7,3	8			8,2		7,1	141	
F044	524	3-4			86	100			83		391	9,5	136			3,4		7,42	134	
F068	678	<3		38	140	90			81		117	4,8	82			7,4		7,36	137	
F018	101	3-4		37,9	147	123			137		221	6,9	28			3,1		7,19	138	
F026	52	<3		36,7	129	73			316		60	1,2	49			2,7		7,16	137	
F014	978	3-4		37,9	120	77			190		143	5,5	128			10		7,36	136	
F005		3-4		34	90	97			77		148	4,9	78			8,5		6,98	122	
F050	363	3-4		37	111	76			63		232	5,3	60			9,3		7,43	133	
F042	93	3-4		38	140	73			88		215	9,7	27			3,1		7,3	148	
F037	601	3-4		34,8	112	82			137		404	13,9	55			10		7,16	127	
F066	855	3-4		36,2	120	73			153		219	5,5	77			9,3		7,04	135	
F010	153	3-4		35,9	110	70			162		107	7,3	12			3,7		7,46	140	
F015	679	3-4		36,9	112	109			75		55	4,6	192			9,7		7,52	130	
F036	605	<3		34,5	105	113			66		253	4,2	48			5,7		7,13	136	
F070	365	3-4		37	123	57			187		161	4,6	68			8		7,35	133	
F048	300	3-4		37,1	114	60			73		335	7,5	62			10		7,38	130	
F049	82	<3		38,1	130	71			162		93	3,2	31			2,7		7,3	137	
F001		3-4		36,5	80	85			94		466	6,7	97			10		7,43	132	
F020	695	3-4		37,2	131	62			173		132	10,7	54			3,5		7,21	140	
F041	149	3-4		36,8	90	100			240		53	1,4	492			6		7,48	139	
F039	699	<3		37	100	107			242		433	13,3	64			6,7		7,33	131	
F063	89	<3		36,5	105	60			22		134	8,9	76			2,7		7,37	138	
F029	155	<3		37,2	116	70			194		302	8,2	58			7,3		7,31	144	
F013	107	<3		39	120	67			53		127	3,5	68			6,7		7,45	136	
F061	134	<3		37	120	86			210		141		82			11		7,42	137	
F064	94	<3		36,1	160	67			278		69	2,6	139			7		7,24	138	
F007	221	<3		36,8	115	50			52		79	8,7	47			9,3		7,48	134	
F024	107	<3		36,8	110	80			34		57	1,2	117			5,7		7,41	137	
F054		3-4		36,7	69	76			239		65	2,5	86			2,9		7,42	138	
F055	474	3-4		35,4	90	85			106		151	5,9	50			10		7,51	143	
F043	391	<3		36,9	88	70			122		55	4,4	60			5,3		7,46	130	
F032	270	3-4			111	67			376		50	3,2	96			6,5		7,41	139	
F067	313	3-4		37	92	95			98		75	12,4	80			6,7		7,52	137	
F023		3-4		37	85	91			367		60	2,9	76			4,7		7,4	137	
F060	557	3-4		36	60	93			76		135	5,1	50			6,6		7,37	137	
F009	84	<3		36,5	85	113			148		60	7,6	41			3,9		7,41	126	
F006	56	<3		37,1	70	85			123		56	1,5	83			3,6		7,44	139	
F017	50	<3		37,5	89	102			168		49	1,3	50			3,8		7,4	136	

Patient code	HCO3 (mmol/L)	BE (mmol/L)	Lactate (mmol/L)	pCO2 (kPa)	PaO2 (kPa)	FiO2 (%)	PaO2/FiO2 (kPa)	A-a gradient (mmHg)	Mechanical ventilation	PEEP	Vasopressor	Noradrenaline dose	Renal replace therapy	SIRS adm	SIRS	MELD	UKELD	iMELD	APACHE2	SOFA	KCH criteria at admission	CLIF-OF	CLIF ACLF grade	CLIF ACLF score	CLIF AD	ALF-Score
P0067	16,1	-10,5	14,2	5,65	14,57	70	20,8	344,3	yes	10	yes	93,3	yes	3	yes	34,7	66	8	34	19	y	16	3	65	89	8,1
P0024	12,2	-18,7	9,8	6,74	13,18	100	13,2	563,6	yes	11	yes	130	no	2	yes	36,6	77	8	28	16	y	14	3	58	96	8,1
P0124	18,3	-9,3	19,2	6,3	24,2	100	24,2	484,3	yes	15	yes	66,6	yes	3	yes	38,8	77	9	37	19	y	16	3	66	104	7,6
P0156	15,4	-11,1	5,9	4,5	7,9	50	15,8	259,3	yes	6	yes	54	yes	2	yes	36,5	77	8	36	18	y	16	3	62	95	7,3
P0055	15,6	-7,4	14,3	2,94	7,24	40	18,1	205,5	yes	11	yes	72	yes	4	yes	33,5	69	7	25	12	y	15	3	69	95	7,3
P0078	21,6	-6,5	8,2	7,46	14,64	50	29,3	183,8	yes	5	yes	66,6	yes	2	yes	35,7	68	9	38	15	y	15	3	69	92	7,2
P0092	20,1	-2,5	9,7	4,77	9,64	45	21,4	207,9	yes	6,2	yes	26,66	yes	3	yes	39,9	81	9	34	20	y	17	3	46	74	7,2
P0061	9,3	-18,1	14,6	3,54	21,6	100	21,6	524,5	yes	13,6	yes	37	yes	2	yes	33	67	8	31	14	y	16	3	57	75	7,0
P0152	22,1	-3,6	7,3	5,7	10,4	55	18,9	266,6	yes	5	yes	13,33	yes	2	yes	32,8	69	8	35	20	y	17	3	64	75	6,9
P0072	21,6	-2,2	13,1	4,33	13,85	30	46,2	71,9	yes	6,5	yes	64	yes	2	yes	36,1	64	8	29	15	y	14	3	54	81	6,8
P0046	19,8	-7,9	5,7	4,02	17,43	100	17,4	552,1	yes	10	yes	5,3	yes	3	yes	38,6	71	8	32	16	y	17	3	62	89	6,8
P0178	11,3	-17,4	35,8	5,33	26,4	80	33	330,4	yes	5	yes	42,6	yes	2	yes	28,5	65	7	37	13	y	15	3	57	77	6,7
P0115	19	-8,1	5,1	5,3	21,1	30	70,3	8,9	yes	4,9	yes	21,33	yes	3	yes	43	81	9	36	17	y	16	3	57	90	6,7
P0138	16,6	-11,1	7,1	6	10,9	60	18,2	296,6	yes	7,5	yes	40	no	2	yes	32,7	66	9	37	16	y	15	3	75	102	6,7
P0136	28,8	5	4	6,1	11,1	30	37	76,9	yes		yes	20	no	2	yes	42,2	70	9	31	18	y	16	3	52	83	6,7
P0129	12,1	-13,4	19,2	3,9	12,8	35	36,6	119,5	yes	5,5	yes	38,6	yes	3	yes	35,4	72	8	22	12	y	15	3	57	86	6,6
P0071	23,4	-4,6	4,5	7,95	10,03	60	16,7	287	yes	9,3	yes	34,6	yes	2	yes	31	65	6	34	17	y	15	3	63	87	6,6
P0113	13,3	-12,7	8,8	4,3	36,7	50	73,4	45	yes	5,4	yes	53,33	yes	4	yes	33,8	65	8	26	12	y	14	3	62	90	6,5
P0154	15,8	-11,3	6,5	5	12,6	40	31,5	147,6	yes	5	yes	33,3	yes	3	yes	36,4	70	9	31	13	y	15	3	64	90	6,5
P0120	9,3	-21,5	16,9	5,13	33,05	30	110,2	-79,2	yes	4,8	yes	49,5	yes	3	yes	35,3	72	9	34	14	y	14	3	63	100	6,5
P0128	21,7	-4,3	5,3	6,6	15,7	60	26,2	255,6	yes	8,3	yes	9,33	yes	2	yes	29,5	73	8	35	16	y	16	3	70	99	6,4
P0062	17,7	-7,7	4,7	4,12	14,27	50	28,5	214,7	yes	5,6	yes	8	yes	1	no	28,7	71	8	25	13	y	16	3	64	79	6,4
P0109	12,4	-15,3	16,1	3	13,6	40	34	157,3	yes	7,5	yes	24	yes	4	yes	25,3	72	6	30	11	y	15	3	65	76	6,3
P0103	16	-6,9	4	4,57	14,43	40	36,1	137,6	yes	8,4	yes	4	yes	3	yes	37,9	77	9	28	11	y	16	3	66	100	6,3
P0082	19,5	-5,4	10	5,01	13,71	50	27,4	211,4	yes	10,1	yes	21,3	yes	2	yes	37	75	7	25	9	y	15	3	51	77	6,3
P0191	12,4	-13,3	3,2	4,02	19,5	30	65	32,2	yes	5	yes	0	yes	2	yes	42,4	74	9	36	18	y	16	3	60	90	6,3
P0166	17,4	-7,2	3,3	4,14	13,5	40	33,8	148,2	yes		yes	0	yes	4	yes	44,1	79	10	24	13	n	16	3	70	109	6,3
P0133	21,3	-9,2	20,3	8,7	10,9	85	12,8	456,6	yes	10,4	yes	0	yes	4	yes	42	77	9	33	13	y	16	3	48	82	6,3
P0006	12,4	-14,4	5,9	4,33	11,26	40	28,2	163,4	yes	7	yes	16	yes	2	yes	36,1	69	8	22	13	y	15	3	60	88	6,2
P0118	22	-1,2	9,5	4,5	16,8	30	56	48,2	yes	5	yes	13,3	yes	3	yes	33,5	71	8	32	13	y	15	3	58	79	6,1
P0027	25,1	-0,2	3,1	6,33	14,17	50	28,3	196,8	yes	5	yes	10,6	no	3	yes	27,9	55	8	23	12	n	15	3	69	65	6,1
P0104	21,3	-3	3,8	4,6	12,16	30	40,5	82,2	yes	6,1	yes	2,7	yes	2	yes	39	71	9	32	14	y	15	3	54	83	5,9
P0169	25	0,8	5,7	5,2	14,6	40	36,5	130,9	yes		yes	0	yes	3	yes	33,2	68	8	31	12	y	15	3	66	92	5,9
P0194	26,4	1,2	4,2	4,59	9,8	60	16,3	316,4	yes	2,6	yes	0	no	3	yes	35,3	70	8	25	17	y	15	3	59	80	5,9
P0117	13,4	-14,7	14,5	3,5	6,3	40	15,8	207,8	no		no	0	yes	4	yes	38,6	73	7	30	12	y	15	3	52	78	5,9
P0097	16,3	-8,7	8,3	4,83	19,8	35	56,6	58,9	yes	5,1	yes	16	yes	3	yes	35,4	75	8	26	10	y	14	3	57	96	5,8
P0163	21,2	-0,4	4,2	4	13,1	21	62,4	15,6	yes	2	yes	13,3	yes	3	yes	44,1	75	9	25	12	y	14	3	54	101	5,7
P0183	16,3	-9,1	5	4,63	26,8	40	67	44,3	yes		yes	9,1	yes	3	yes	36	73	8	30	12	y	14	3	57	96	5,7
P0116	13,1	-14,6	5,5	3,9	19,71	30	65,7	31,7	yes	5	yes	26,6	no	3	yes	37,6	77	8	24	12	y	13	3	53	92	5,6
P0110	24,9	1,3	2,6	5	16,4	50	32,8	191,3	yes	5,2	yes	6,6	no	2	yes	35,5	76	8	19	10	n	14	3	47	83	5,6
P0019	17,7	-7,5	3,4	4,1	18,2	30	60,7	41,3	yes	6,2	yes	5,33	yes	2	yes	24,5	60	7	14	8	n	14	3	60	74	5,6
P0059	18,3	-6,8	2,4	4,68	17,46	28	62,4	27,3	yes	6,2	yes	5,3	yes	3	yes	36,5	71	9	33	12	n	14	3	66	98	5,6
P0200	16	-18		2	33,4	50	66,8	89,1	yes		yes	0	yes	4	yes	33,8	63	8	33	12	y	14	3	53	78	5,5
P0028	18	-7,4	1,3	4,66	18	30	60	37,8	yes	6,5	no	0	yes	1	no	29,6	74	8	27	13	n	14	3	53	75	5,5
P0162	21,7	-1,8	3,3	4,3	16,8	30	56	50	yes	5	yes	0	yes	4	yes	36,1	72	9	35	13	n	14	3	59	87	5,5
P0167	21,8	-0,2	2,8	4,27	10,5	24	43,8	54,3	no		yes	0	yes	2	yes	38,9	70	8	15	13	n	14	3	67	96	5,5
P0197	10,7	-13,2	6	2,77	11,2	21	53,3	40,9	no		yes	0	yes	3	yes	37,5	77	9	41	14	y	14	3	60	99	5,5
P0157	14,8	-12,2	1,5	4	12	50	24	232,8	yes	5	yes	0	yes	3	yes	15,1	58	6	26	13	y	14	3	57	61	5,5
P0143	25,6	-0,7	2,9	6,8	20	100	20	512	yes	7,8	no	0	yes	2	yes	36,9	78	8	25	12	y	14	3	60	104	5,5
P0083	13,5	-11,5	11,5	3,84	31,53	50	63,1	87,6	yes	8,8	yes	8	no	2	yes	36,2	71	9	31	14	y	13	3	61	97	5,2
P0123	22,2	-0,2	5,4	4,1	11,5	21	54,8	26,7	no		yes	8	yes	3	yes	33,7	73	7	18	10	y	13	3	60	95	5,2
P0085	21,5	-1,4	1,4	4,3	11,26	40	28,2	163,7	yes	6,3	yes	6,6	no	2	yes	21,2	61	7	26	10	n	13	3	52	57	5,2
P0029	20,4	-4,7	5,9	4,89	11,17	28	39,9	72,6	yes	11	yes	5,3	yes	2	yes	17,6	57	6	25	12	y	13	3	42	47	5,2
P0146	13,4	-16,5	10,9	6,4	12,3	30	41	65,3	yes	5,4	yes	4	no	4	yes	37,1	69	8	27	13	y	13	3	50	83	5,2
P0089	23,6	1,6	2,8	4	22	30	73,3	13,7	yes	5,5	yes	4	no	4	yes	39,3	63	8	17	13	n	13	3	41	62	5,2
P0170	23,1	-0,7	2,9	4,73	14,9	21	71	-4,5	yes		yes	0	no	4	yes	37,4	68	8	23	11	n	13	3	51	81	5,1
P0094	10,9	-12,3	3,4	5,58	16,23	30	54,1	43	yes	4,5	no	0	yes	3	yes	36,4	76	8	26	10	y	13	3	45	87	5,1
P0159	18,8	-5,8	2,8	4,7	17,2	30	57,3	43,5	yes	4,5	no	0	yes	2	yes	28,2	74	8	30	13	n	13	3	51	71	5,1
P0192	18,2	-3,8	2,8	3,99	14,5	21	69	5,1	no		yes	0	yes	2	yes	37,1	77	6	20	10	n	13	3	43	87	5,1
P0056	14,3	-11,6	3,7	4,09	16,38	40	41	127,1	no		no	0	yes	2	yes	31,2	82	9	27	9	y	13	3	60	92	5,1
P0172	11,4	-12,3	11,1	3,13	17,1	21	81,4	-6,6	no		yes	0	yes	3	yes	34,5	70	6	19	10	y	13	3	53	93	5,1
P0181	7	-23,4	13,7	3,8	22	44	50	116,2	no		no	0	yes	3	yes	34,6	70	6	24	11	y	13	2	44	76	5,1
P0190	13,9	-10,7	2,5	3,76	24,1	35	68,9	36	no		yes	0	yes	3	yes	37,8	69	7	19	10	y	13	3	54	93	5,1
P0141	16	-7,1	5,2	3,48	19,31	30	64,4	38,4	yes	5,2	yes	4	no	3	yes	40,7	75	9	25	10	y	12	3	56	104	4,8
P0100	22,6	-0,4	2,5	4,3	18,28	30	60,9	38,9	no		no	0	yes	4	yes	37,8	71	6	16	8	n	12	2	47	88	4,7
P0142	25,2	1,1	3,7	5,5	24,5	50	49	126,3	yes	5,2	no	0	yes	4	yes	36,6	74	9	20	10	y	12	3	53	97	4,7
P0068	21,4	-1,9	3	4,78	8,91	28	31,8	90,5	yes	5,1	no	0	no	1	no	25	57	7	22	9	n	12	2	36	43	4,7
P0135	16,5	-6,2	3,1	3,6	12,6	21	60	22,9	no		no	0	yes	4	yes	30,6	75	8	20	8	n	12	3	43	77	4,7
P0177	23	-0,9	2,5	4,14	13,6	21	64,8	10,5	yes	4	yes	0	no	4	yes	35,8	68	8	15	7	n	12	3	46	79	4,7
P0026	20,6	-2,4	4	4,15	12,48	21	59,4	18,9	no		no	0	yes	3	yes	30,5	69	7	22	11	y	12	2	53	80	4,7
P0132	19,7	-2,6	4,8	3,8	13,1	21	62,4	17,4	no		no	0	yes	3	yes	38,1	81	7	21	9	y	12	2	48	99	4,7
P0160	20,4	-3,6	12,1	5	25,5	40	63,8	50,8	yes	5	no	0	no	4	yes	24	65	7	23	12	y	12	3	55	82	4,7
P0121	13,7	-10,4	8,9	3,53	13,66	28	48,8	65,9	no		no	0	yes	3	yes	39	82	7	21	10	y	12	2	53	102	4,7
P0106	14,4	-3	3,8	3,58	13,31	21	63,4	17,8	no		no	0	yes	3	yes	32,4	70	6	16	8	y	12	2	38	67	4,7
P0130	23	1,9	6,3	4	13,7	21	65,2	11,1	no		no	0	yes	3	yes	36,4	77	7	18	10	y	12	2	48	86	4,7
P0030	11	-16,8	7,6	4,01	13,87	35	39,6	110,6	yes	6,9	yes	10,6	no	3	yes	10	45	6	24	6	y	11	2	57	51	4,5
P0122	20	-5,4	8,9	2,7	10,3	21	49	48,2	no		yes	6,6	no	3	yes	29	65	6	9	6	y	11	2	40	72	4,4
P0147	32,2	2,4	3,6	7	14,5	30	48,3	43,5	yes	4,9	no	0	no	3	yes	42,7	70	9	19	9	y	11	2	46	95	4,3
P0140	18,1	-5,4	1,6	4,2	14,3	30	47,7	69,6	yes	5,1	no	0	no	3	yes	20,2	75	7	26	10	n	11	2	41	72	4,3
P0093	18,3	-3,9	0,8	3,41	11,66	21	55,5	31,7	no		no	0	yes	2	yes	27,8	73	6	20	8	n	11	2	46	88	4,3
P0095	23,7	2,8	2,1	5,3	21,3	40	53,3	79,7	yes	5	no	0	no	1	no	28,5	61	7	15	7	n	11	2	40	61	4,3
P0137	34,6	10,8	2,5	5,6	11,2	21	53,3	15,4	no		no	0	no	2	yes	30,5	68	6	13	7	n	11	2	36	66	4,3
P0070	26,9	2,7	3,2	5,54	20,97	21	99,9	-57,3	no		no	0	no	2	yes	42,7	70	7	9	6	n	11	2	48	80	4,3
P0090	18,8	-6,9	5,4	4,93	11,3	60	18,8	302,4	no		no	0	no	4	yes	29,8	60	7	17	10	y	11	2	42	62	4,3
P0125	28,4	6,9	5	4,5	12,8	21	61	13,3	no		no	0	yes	2	yes	34,2	68	6	8	4	y	11	2	44	83	4,3
P0076	17,5	-5,9	8,1	3,74	11,71	21	55,8	28,3	no		no	0	no	3	yes	35,8	66	9	11	3	y	10	2	48	81	3,9
P0080	22,3	-2,6	1,2	5,24	13,72	30	45,7	64,8	yes	5,1	no	0	no	0	no	24,6	59	7	18	9	n	10	1	36	51	3,9
P0112	25	1,6	2,4	4,4	11	21	52,4	27,7	no		no	0	no	0	no	37,2	75	7	11	4	n	10	1	41	87	3,9
P0074	20	-3,2	2,4	3,93	10,7	21	51	34,2	no		no	0	no	1	no	24,1	64	5	12	6	n	10	2	38	68	3,9
P0155	25,4	0	1,5	4,8	12,1	21	57,6	15,9	no		no	0	no	3	yes	34,8	65	6	6	5	n	10	1	40	73	3,9
P0038	24,6	1,4	5,3	4,73	13,38	21	63,7	6,9	no		no	0	no	1	no	33,6	70	6	9	4	y	10	1	38	72	3,9
P0114	21,8	-4,4	5,2	5,4	11,9	30	39,7	77,1	yes	4,8	no	0	no	2	yes	12,6	57	7	29	4	y	10	no	0	59	3,9
P0009	20,8	-2,1	5	3,96	21	27	77,8	-0,1	no		no	0	no	3	yes	35,4	64	6	11	4	y	10	1	43	75	3,9
P0034	22,1	-2,8	2,9	5,16	36,55	60	60,9	111,1	yes	6,5	no	0	no	2	yes	8	45	5	23	5	n	9	no	0	41	3,5
P0158	28,7	4,6	1,8	5,6	13,5	21	64,3	-1,8	no		no	0	no	2	yes	26,8	63	5	4	4	n	9	1	32	62	3,5
P0048	21,1	-2,5	3,8	4,52	12,83	21	61,1	12,9	no		no	0	no	1	no	28,8	62	6	6	3	y	9	1	37	69	3,5
P0011	23,4	0,8	3	4,12	10,5	21	50	34	no		no	0	no	2	yes	33,4	65	6	8	4	y	9	1	35	75	3,5
P0037	18,7	-4,9	4	4,06	20,21	21	96,2	-38,3	no		no	0	no	2	yes	33,7	66	6	10	4	y	9	1	35	73	3,5
F025	24		10,6	4,27	11,73	80	14,7	448,8	yes		yes	265,6	yes									17				12,0
F019	17		6,5	2,8	10,93	50	21,9	250,9	yes		yes	199,2	yes									18				11,0
F058			19,9		11,2	100	11,2	629	yes		yes	215,8	yes									17				11,0
F012	7		20	3,33	17,07	100	17,1	560	yes		yes	232,4	yes									16				10,9
F008	11		10,6	2,67	9,87	60	16,4	331,8	yes		yes	182,6	yes									16				9,9
F059			24		31,73	21	151,1	-88,3	no		yes	182,6	yes									14				9,1
F038			22,5		18,67	80	23,3	430,4	yes		yes	131,1	yes									16				8,9
F021	10		16,7	3,6	8,27	100	8,3	624	yes		yes	78	yes									18				8,6
F003	12		24,9	3,73	17,07	50	34,1	197	yes		yes	132,8	yes									15				8,5
F035	16		9,1	4,8	20,8	66	31,5	275,5	yes		yes	132,8	yes									15				8,5
F033	18		11,5	4,13	13,2	55	24	258,7	yes		yes	83	yes									16				7,9
F030	19		2,5	3,73	11,2	21	53,3	32,2	no		yes	83	yes									16				7,9
F053			3		10,27	100	10,3	636	yes		yes	89,6	yes									15				7,7
F011	15		16	6	13,87	40	34,7	129,5	yes		yes	83	yes									15				7,5
F002	19,1		6	4,25	11,47	100	11,5	595,1	yes		yes	58,1	yes									16				7,4
F028	7		15,4	3,87	35,33	50	70,7	58,9	yes		yes	91,3	yes									14				7,3
F047	7		13,2	3,87	15,07	45	33,5	174,9	yes		yes	66,4	yes									15				7,2
F044	23		10,2	3,33	30	90	33,3	391,1	yes		yes	44,8	yes									16				7,2
F068	23		12,7	4,93	21,87	21	104,1	-58,6	no		yes	116,2	no									12				7,0
F018	13		9,3	4,53	25,33	100	25,3	489	yes		yes	33,2	yes									16				6,9
F026	6		12,3	1,33	8	21	38,1	77,8	no		yes	43,2	yes									15				6,7
F014	16		11	3,87	14,4	21	68,6	7	yes		yes	33,2	yes									15				6,5
F005	6		23,5	2,8	15,2	100	15,2	578	yes		no	44,8	yes									14				6,4
F050	13		8,9	3,2	10,8	21	51,4	40	yes		no	83	yes									12				6,4
F042	16		5,5	4,93	6,93	80	8,7	479,6	yes		yes	16,6	no									15				6,2
F037	14		4,8	6	9,07	50	18,1	237,9	yes		no	13,3	yes									14				5,7
F066	4		18,2	2,13	10,27	21	48,9	53,6	no		yes	3,3	yes									14				5,5
F010	19		2,9	5,47	20,53	40	51,3	84,1	yes		yes	28,2	no									12				5,3
F015	22		9,8	3,6	10,53	21	50,2	38,4	yes		yes	6,6	no									13				5,2
F036	7		15,2	2,4	25,73	21	122,5	-64,8	no		no	41,5	no									11				5,1
F070	12		12,4	2,93	9,87	21	47	49,4	no		no	0	yes									13				5,1
F048	22		2,4	5,07	12	21	57,1	14,2	yes		no	0	yes									13				5,1
F049			5,6		25,33	21	120,6	-40,3	no		no	0	yes									12				4,7
F001	23		3,2	4,53	20,27	30	67,6	22	yes		no	0	yes									12				4,7
F020	9		13,3	2,93	16,4	21	78,1	0,4	no		no	0	yes									12				4,7
F041			2,3		31,73	21	151,1	-88,3	yes		no	0	no									12				4,7
F039			5,1		31,73	21	151,1	-88,3	no		no	0	yes									12				4,7
F063			3,9		25,33	21	120,6	-40,3	no		no	0	no									11				4,3
F029			23		25,33	21	120,6	-40,3	no		no	0	no									11				4,3
F013			8,3		31,73	21	151,1	-88,3	no		no	0	no									11				4,3
F061	16		4,4	3,2	12,93	21	61,6	24	no		no	0	no									11				4,3
F064			9,6		31,73	21	151,1	-88,3	no		no	0	no									11				4,3
F007	19		3	4	16,53	21	78,7	-10,2	no		no	0	no									11				4,3
F024	20		2,6	4,13	14	21	66,7	7,6	no		no	0	no									10				3,9
F054	19		2,7	3,87	15,6	21	74,3	-2	no		no	0	no									10				3,9
F055	24		5,3	5,33	23,2	50	46,4	137,5	yes		no	0	no									10				3,9
F043	20		5	3,73	11,07	21	52,7	33,2	no		no	0	no									10				3,9
F032	13		3,1	3,73	18,27	21	87	-20,8	no		no	0	no									10				3,9
F067	23		2	3,73	23,47	35	67	41	yes		no	0	no									10				3,9
F023	17		3,6	4,13	14,8	21	70,5	1,6	no		no	0	no									9				3,5
F060			3,8		31,73	21	151,1	-88,3	no		no	0	no									9				3,5
F009			4,1		31,73	21	151,1	-88,3	no		no	0	no									9				3,5
F006	18		1,3	4,13	14,4	21	68,6	4,6	no		no	0	no									9				3,5
F017	21		1,1	4	12,67	21	60,3	18,8	no		no	0	no									9				3,5
